# Clemastine fumarate accelerates accumulation of disability in progressive multiple sclerosis by enhancing pyroptosis

**DOI:** 10.1172/JCI183941

**Published:** 2025-05-15

**Authors:** Joanna Kocot, Peter Kosa, Shinji Ashida, Nicolette A. Pirjanian, Raphaela Goldbach-Mansky, Karin Peterson, Valentina Fossati, Steven M. Holland, Bibiana Bielekova

**Affiliations:** 1 Neuroimmunological Diseases Section, Laboratory of Clinical Immunology and Microbiology, National Institute of Allergy and Infectious Diseases, NIH, Bethesda, Maryland, USA.; 2 The New York Stem Cell Foundation Research Institute, New York, New York, USA.; 3 Translational Autoinflammatory Diseases Section, Laboratory of Clinical Immunology and Microbiology, National Institute of Allergy and Infectious Diseases, NIH, Bethesda, Maryland, USA.; 4 Neuroimmunology Section, Laboratory of Neurological Infections and Immunity, Rocky Mountain Laboratories, National Institute of Allergy and Infectious Diseases, NIH, Hamilton, Montana, USA.; 5 Immunopathogenesis Section, Laboratory of Clinical Immunology and Microbiology, National Institute of Allergy and Infectious Diseases, NIH, Bethesda, Maryland, USA.

**Keywords:** Immunology, Neuroscience, Cellular immune response, Multiple sclerosis, Neurodegeneration

## Abstract

Multiple sclerosis (MS) is an immune-mediated demyelinating disease of the CNS. Clemastine fumarate, the over-the-counter antihistamine and muscarinic receptor blocker, has remyelinating potential in MS. A clemastine arm was added to an ongoing platform clinical trial, targeting residual activity by precision, biomarker-guided combination therapies of multiple sclerosis (TRAP-MS) (ClinicalTrials.gov NCT03109288), to identify a cerebrospinal fluid (CSF) remyelination signature and to collect safety data on clemastine in patients progressing independently of relapse activity (PIRA). The clemastine arm was stopped per protocol-defined criteria when 3 of 9 patients triggered individual safety stopping criteria. Clemastine-treated patients had significantly higher treatment-induced disability progression slopes compared with the remaining TRAP-MS participants. Quantification of approximately 7,000 proteins in CSF samples collected before and after clemastine treatment showed significant increases in purinergic signaling and pyroptosis. Mechanistic studies showed that clemastine with sublytic doses of extracellular adenosine triphosphate (ATP) activates inflammasome and induces pyroptotic cell death in macrophages. Clemastine with ATP also caused pyroptosis of induced pluripotent stem cell–derived human oligodendrocytes. Antagonist of the purinergic channel P2RX7, which is strongly expressed in oligodendrocytes and myeloid cells, blocked these toxic effects of clemastine. Finally, reanalysis of published single-nucleus RNA-Seq (snRNA-Seq) studies revealed increased P2RX7 expression and pyroptosis transcriptional signature in microglia and oligodendrocytes in the MS brain, especially in chronic active lesions. The CSF proteomic pyroptosis score was increased in untreated MS patients, was higher in patients with progressive than relapsing-remitting disease, and correlated significantly with the rates of MS progression. Collectively, this identifies pyroptosis as a likely mechanism of CNS injury underlying PIRA even outside of clemastine toxicity.

## Abstract 2

<a>Clemastine fumarate accelerates accumulation of disability in progressive multiple sclerosis by enhancing pyroptosis </a>

## Introduction

Multiple sclerosis (MS) is a chronic immune-mediated demyelinating disease of the central nervous system (CNS). Initial stages of MS are characterized by formation of focal inflammatory CNS lesions called MS plaques. Some MS plaques lead to subacute onset of neurological disability called MS relapse. All current disease-modifying treatments (DMTs) target this focal inflammatory “MS lesional activity” (i.e., MS plaques and relapses) very effectively; newer treatments inhibit formation of MS lesions by more than 95%. Despite this, the DMTs inhibit disability progression only modestly. In a metaanalysis of randomized clinical trials (1) DMTs initiated after a mean age of 30 years do not inhibit disability progression completely, and their efficacy decreases linearly with the patient’s age. After a mean age of 53 years, DMTs do not inhibit disability progression beyond placebo. Based on MS prevalence (2, 3), this means that 45% of people living with MS today have no effective treatments and an additional more than 40% of people with MS continue to accumulate disability on current DMTs.

Recognizing this unmet need, MS drug development focuses on targeting new disease mechanisms. Among these is remyelination, as chronically demyelinated axons are considered less resilient to neurodegenerative mechanisms compared with their myelinated counterparts (4). Clemastine (CLM) fumarate, an over-the-counter antihistamine with anticholinergic properties against muscarinic M1 receptor, induces remyelination in vitro (5), in multiple animal models (6–9), and in a randomized, blinded cross-over phase II MS trial (ReBUILD; ClinicalTrials.gov NCT02040298) (10). In 50 MS patients with a mean age of 40.1 years who had mild disability (i.e., mean Expanded Disability Status Scale [EDSS] 2.2 on ordinal scale from 0 to 10) (11), CLM demonstrated a remyelinating effect. The primary outcome was drug-induced acceleration of the electrical conduction across visual pathways measured by the change in latency of the visual evoked potential–positive (VEP-positive) 100 msec wave (P100). MS patients with abnormally prolonged P100 (mean P100 = 128.6 and 126.8 msec in 2 crossover groups) were enrolled. CLM-treated patients decreased P100 latency by 1.7 msec per eye (93 eyes total; *P* = 0.0048). Interpreting measured mean P100 velocities as representing 28.6 to 26.8 msec delay due to MS, CLM corrected on average 5.9%–6.3% of this preexisting abnormality. This was associated with a trend for low contrast visual acuity improvement by the mean of 0.9 letters/eye (*P* = 0.085). CLM also improved brain myelin water fraction, measured as reduction in radial diffusivity from diffusion tensor MRI, but did not affect another MRI biomarker of demyelination, magnetization transfer ratios (12). No significant changes in clinical outcomes were observed in this short trial. None of the clinical trials testing other remyelinating agents showed clear efficacy (13).

The vulnerability of demyelinated axons to degeneration is not the only candidate mechanism of progressing independently of relapse activity (PIRA). Analyses of MS CNS tissue (14, 15) and cerebrospinal fluid (CSF) biomarkers from MS patients identified compartmentalized inflammation in CNS tissue (16, 17) insufficiently targeted by current DMTs (18) and varied neurodegenerative processes (19–21). These candidate mechanisms evolve with MS duration (22) and, analogous to the heterogeneity of the formation of acute MS lesions (23), are not uniformly detected in all MS patients. This observed heterogeneity of MS disease mechanisms implies that, similarly to other chronic diseases with complex pathophysiology such as cardiovascular diseases, patient-specific “custom combination treatments” may be required to inhibit disability progression in MS. The prerequisite for development of such personalized treatments is the ability to measure candidate disease mechanisms comprehensively in living people. To achieve this goal, in 2017 we initiated a platform, open-label, CSF biomarker–guided clinical trial: targeting residual activity by precision, biomarker-guided combination therapies of multiple sclerosis (TRAP-MS; ClinicalTrials.gov NCT03109288). The objectives of TRAP-MS are the following: (a) to develop clinical trial methodology that allows economical screening of prospective therapeutic agents for their efficacy on biological processes related to MS severity using CSF biomarkers; (b) to develop a knowledge base of intrathecal effects of current DMTs and new treatments targeting varied mechanisms of MS progression; and (c) to establish and validate a framework for development of effective combination therapies for MS.

Based on the results of the ReBUILD trial, in 2020 we added a CLM arm to the TRAP-MS trial with the goal of identifying CSF remyelination signature and extending the safety/tolerability data on the long-term use of CLM in patients progressing by PIRA (i.e., by “nonlesional” MS activity). We stopped the CLM arm in 2022 due to protocol-defined safety criteria. This paper identifies CLM-enhanced pyroptosis as the mechanism for observed toxicity. Most importantly, we show that pyroptosis associated with smoldering intrathecal inflammation is likely the mechanism of PIRA in general.

## Results

### CLM arm of TRAP-MS protocol was stopped after 3 out of 9 patients triggered safety-stopping criterion.

**Figure 1:**
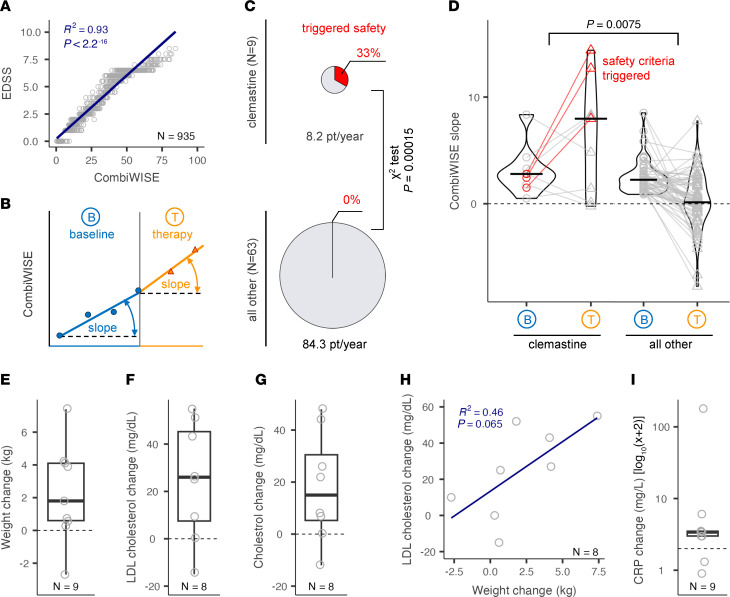
CLM-induced changes in disability, metabolism, and inflammatory markers. (**A**) Clinical safety was monitored by a continuous, machine-learning derived CombiWISE scale (range 0–100) that correlates strongly with EDSS (range 0–10). R-squared in Linear Regression was used. *n* = 935. (**B**) A minimum of 4 visits on stable therapy spanning at least 18 months is required to measure baseline (blue) CombiWISE slope. On-therapy CombiWISE slope (orange) is calculated based on 6-months follow-up data collected after therapy initiation. (**C**) Three out of 9 patients on CLM therapy triggered safety criteria, in contrast to none of the 63 patient-specific treatments of 6 other TRAP-MS therapies; *P* value of probability of this occurrence was based on χ^2^ test. (**D**) Progression CombiWISE slopes at baseline (B) and therapy (T) were compared between CLM arm (*n* = 9) and all other 6 TRAP-MS therapies (*n* = 63). Black horizontal line represents median value of the group. Red color indicates 3 patients that triggered safety-stopping criteria treatment slope exceeding 5× baseline slope. Displayed *P* value was generated from 2-sided unpaired Wilcoxon’s rank test comparing therapy-induced change in CombiWISE slope between the CLM arm and all other therapies. CLM induced increase of weight (**E**), LDL cholesterol (**F**), and total cholesterol (**G**) levels between baseline and treatment and these changes showed strong association as seen on example of weight change versus LDL cholesterol change (**H**). R-squared in linear regression was used. Furthermore, CLM induced increase of inflammatory biomarker CRP (**I**). Lipid panel was an optional laboratory test, and the results are missing for 1 patient. Blue line in **H** represents linear regression line; gray dashed line in **D**–**G** and **I** represents 0 change. The lower and upper hinges of the boxplots correspond to the first and third quartiles (the 25th and 75th percentiles). The upper and lower whiskers extend from the hinge to the largest and smallest value, respectively.

TRAP-MS patients are monitored with clinical and imaging safety outcomes every 6 months and with CSF biomarkers 6 months after each therapeutic change. Clinical safety is monitored by a continuous, machine-learning derived Combinatorial Weight Adjusted Disability Scale (CombiWISE; range 0–100) (24). CombiWISE correlates highly with EDSS (R^2^ = 0.93, *P* < 0.0001; Figure 1A) and reproducibly measures yearly disability progression in small (~30 subjects) MS cohorts (24). To receive treatment under the TRAP-MS protocol, patients on stable DMT (or untreated if desired) must be progressing by at least 0.5 CombiWISE units/year, derived from the patient-specific linear regression models based on a minimum of 4 clinic visits with each visit 6 or more months apart (i.e., minimum of 18 months; Figure 1B). This represents a “baseline” progression slope, whereas analogously derived data from on-treatment visits measures “therapy” progression slopes.

TRAP-MS protocol has predefined individual and treatment arm safety-stopping criteria. Individual patients must stop the study drug when the on-treatment yearly CombiWISE progression slope exceeds 5 times the baseline progression slope or when on-treatment accumulation of new/enlarging MS lesions exceeds 3 times the baseline average. Individual stopping criteria were based on natural history cohort simulations from 536 patient/years, where they occur only in 3.4% of all clinic visits. The treatment arm is stopped for toxicity when 3 treated patients trigger individual stopping criteria.

Sixteen patients initiated CLM treatment, with 9 completing at least one 6-month follow-up visit (total of 8.2 patient/years), when 3 of 9 (33.3%) fulfilled individual stopping criteria of on-treatment progression slopes exceeding baseline progression slopes by more than 5-fold (Figure 1C). The probability of seeing this result by chance is 0.015% (*P* = 0.00015; χ^2^ test comparing CLM arm with remaining TRAP-MS treatments, where 0 out of 63 treatment-specific patient slopes [representing 42 patients] triggered safety criteria after 84.3 patient/years on the remaining 6 tested drugs: montelukast, losartan, hydroxychloroquine, pioglitazone, dantrolene and pirfenidone). This triggered per-protocol closing of the CLM arm for toxicity (Supplemental Table 1; supplemental material available online with this article; https://doi.org/10.1172/JCI183941DS1).

Apart from the incidence of patients triggering individual stopping criteria, Figure 1D shows that most (6/9) CLM-treated patients progressed at higher than baseline rates, while most remaining TRAP-MS patients slowed rates of disability progression (*P* = 0.0075 for comparing change in slopes for CLM-treated versus remaining patients). Note that the TRAP-MS trial is ongoing, and no treatment arm reached predefined numbers to analyze clinical outcomes. Therefore, we can’t exclude that the apparent benefit of the remaining drugs is due to the placebo effect universally observed in MS trials.

Based on very low probability that observed results are due to chance, together with vastly different treatment effects on progression slopes between CLM and other TRAP-MS treatments, we conclude that CLM accelerated disability progression in these patients with advanced progressive MS.

### CLM-treated patients developed metabolic syndrome with activation of innate immunity.

The patients who reached stopping criteria tended to be older (median age 71.4 versus 60.6 years), heavier (median weight 93.8 versus 75.5 kg), and more disabled (i.e., median EDSS 7.0 versus 6.5 and median CombiWISE 60.9 versus 52.9) compared with subjects who did not. However, the adverse effects of CLM were seen in most treated patients: we observed an increase in the CombiWISE slope in 67%, weight gain in 89%, increased total cholesterol in 88%, and increased LDL cholesterol in 88% of CLM-treated patients (Figure 1, D–G). These metabolic changes were correlated: e.g., an increase in serum LDL cholesterol explained 46% of variance of weight gain (Figure 1H). Furthermore, 78% of CLM-treated patients increased serum C-reactive protein (CRP) (Figure 1I). These changes were not observed on other TRAP-MS–tested drugs. To summarize, CLM-treated patients exhibited systemic signs of metabolic syndrome and acute phase response suggestive of innate immunity activation.

### Analysis of CSF biomarkers shows that CLM treatment altered purinergic metabolism.

To understand intrathecal effects of CLM, we measured relative concentrations of 7,000 CSF proteins from CSF samples collected before and after CLM therapy using a multiplex DNA-aptamer (SOMAscan) (https://somalogic.com/) assay.

We scaled the measured CSF biomarkers to healthy donor (HD) values (to standardize them for direct comparison) and then calculated changes in biomarker levels between therapy and baseline CSF samples in 9 patients that received CLM therapy. We did not expect any significant differences for individual proteins after adjustment for multiple comparisons because the number of measured proteins greatly exceeded the number of patients/samples, and indeed, none of the CLM-induced changes passed the FDR-adjusted threshold of *P* value of less than 0.05 (Supplemental Table 2).

However, gProfiler analysis of CLM-induced changes in biomarkers with the greatest effect size (those exceeded 2 SDs above and below the mean, representing 5% of biomarkers outside of the normal distribution) unexpectedly identified several significant changes induced by CLM in Gene Ontology (GO) terms related to purine metabolism: e.g., purine nucleotide binding (GO:0017076; *P* = 0.00343), purine nucleotide metabolic process (GO:0006163; *P* = 0.000183), and purine-containing compound biosynthetic process (GO:0072522; *P* = 0.001). We conclude that CLM alters metabolism of purine ribonucleotides.

### CLM potentiates ATP binding to the purinergic P2RX7 receptor and induces activation of inflammasome and pyroptosis in macrophages.

While presenting our unexpected results from the CLM arm of the TRAP-MS trial at an internal research meeting, one of the coauthors (SMH) alerted us to the fact that CLM enhances killing of intracellular pathogens such as mycobacteria in myeloid cells based on its potentiating effect on adenosine triphosphate (ATP) signaling via the purinergic receptor P2X7 (P2RX7) (25, 26). While CLM does not activate P2RX7 by itself, it acts as an allosteric modulator, opening the channel in response to suboptimal/nonactivating concentrations of extracellular ATP.

In immune cells, P2RX7 is highly expressed in myeloid lineage, including microglia (27, 28). P2RX7 activates myeloid cells in response to danger-associated molecular pattern (DAMP) of high extracellular ATP, usually caused by lytic (immunogenic) cell death such as necrosis, necroptosis, or pyroptosis. P2RX7 signaling mediates the efflux of intracellular K^+^, activating the NOD-like receptor pyrin domain-containing protein 3 (NLRP3) inflammasome. Assembled inflammasome activates caspase-1, leading to extracellular release of active IL-1β and IL-18, master regulators of the innate immunity. Inflammasome activation requires 2 signals: (a) priming by pathogen-associated molecular patterns (PAMPs; e.g., lipopolysaccharide [LPS]) or proinflammatory cytokines. Priming initiates transcription of inflammatory mediators, including NLRP3 components and pro–IL-1β. (b) Activation (e.g., by DAMPs such as ATP) leads to inflammasome assembly, resulting in proteolytic cleavage of procaspase-1. Active caspase-1 cleaves other substrates such as gasdermin D (GSDMD), releasing its N-terminal domain (NT-GSDMD) and enabling formation of mitochondrial and plasma membrane channels through which active IL-1β and other inflammatory stimuli are released. Prolonged P2RX7 signaling leads to pyroptotic cell death, further exacerbating inflammation by releasing active caspase-1 and DAMPs such as ATP and nuclear high mobility group box 1 (HMGB1) protein (29, 30).

**Figure 2:**
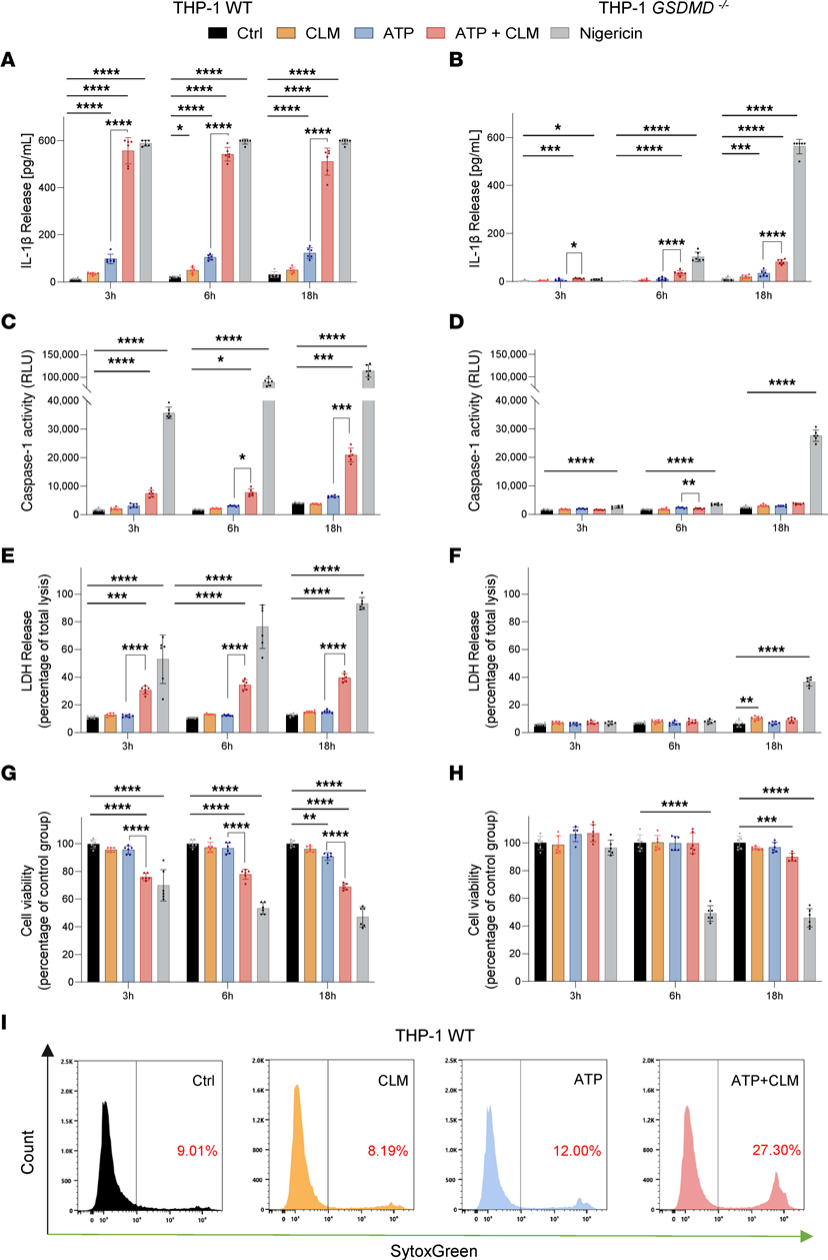
CLM potentiation effect on ATP-induced inflammasome activation and GSDMD-driven pyroptotic cell death in THP-1 cells. The THP-1 cells were primed with 200 ng/mL LPS overnight and then treated either with medium + DMSO (negative control, Ctrl), 10 μg/mL CLM, 2 mM ATP ± 10 μg/mL CLM, or 2.5 μM nigericin (positive control) for 3 hours, 6 hours, and 18 hours. Release of the proinflammatory cytokine IL-1β into the culture medium of THP-1 cells (**A**) and THP-1 *GSDMD^–/–^* cells (**B**), determined by ELISA assay. Activity of caspase-1 in the culture medium of THP-1 cells (**C**) and THP-1 *GSDMD^–/–^* cells (**D**), determined using bioluminescent assay. Lytic cell death of THP-1 cells (**E**) and THP-1 *GSDMD^–/–^* cells (**F**), determined by LDH activity released into the culture medium. Cell viability of THP-1 cells (**G**) and THP-1 *GSDMD^–/–^* cells (**H**) evaluated by the MTS assay. (**A**–**H**) Data are presented as mean ± SD of 3 independent experiments, each performed in duplicate (*n* = 6). One-way ANOVA followed by Dunnett’s multiple-comparisons test was used to compare the testing groups with control group (Ctrl). One-way ANOVA test followed by Holm-Šidák’s multiple comparison was used to compare ATP and ATP+CLM group (to assess the potentiation effect of CLM). *P ≤ 0.05; **P ≤ 0.01; ***P ≤ 0.001; ****P ≤ 0.0001. Panel **A**, ATP+CLM at 3 hours (*n* = 1), nigericin at 3 hours (*n* = 3), 6 hours (*n* = 5), and 18 hours (*n* = 5) — above detection limit of the assay. Panel **B**, Ctrl at 3 hours (*n* = 5) and 6 hours (*n* = 3) — below detection limit of the assay. Nigericin at 18 hours (*n* = 6) — above detection limit of the assay. (**I**) Flow cytometry analysis of cell uptake of SYTOX Green in THP-1 cells primed with 200 ng/mL LPS overnight and then treated either with medium + DMSO (control), 10 μg/mL CLM, or 2 mM ATP ± 10 μg/mL CLM for 90 minutes. Representative plot of 2 independent experiments.

With this background, we asked whether CLM activates inflammasomes (measured by release of IL-1β) in LPS-primed myeloid cells activated by suboptimal ATP concentrations. In the human monocytic leukemia cell line THP-1, used for inflammasome/pyroptosis studies (31), CLM increased release of IL-1β and active caspase-1 (compared with ATP) already 3 hours after activation (Figure 2, A and C, Supplemental Figure 1A, respectively). These effects were mediated by the GSDMD channel, as GSDMD-deficient THP-1 cells released significantly less active caspase-1 (Figure 2D) and IL-1β (Figure 2B). Lack of GSDMD markedly attenuated but did not abolish the potentiating effect of CLM on IL-1β secretion, suggesting that other channels, e.g., gasdermin E (GSDME), may be involved. The above observations were further confirmed by Western blotting. The CLM+ATP treatment markedly increased ATP-induced GSDMD cleavage in THP-1 cells, as evidenced by increased NT-GSDMD expression in cell lysates (Supplemental Figure 2A) and release of both N- and C-terminal fragments to the culture medium (Supplemental Figure 2B). Similarly, we detected protein bands corresponding to active p20 and p33 (CARD-p20) cleaved subunits for caspase-1 in culture medium of ATP-treated cells, further potentiated by ATP+CLM treatment. The lack of GSDMD abolished the release of active caspase-1 (CASP1) subunits into culture medium (Supplemental Figure 2C).

CLM also enhanced immunogenic cell death of THP-1 cells, reflected by the loss of cell membrane integrity measured by cellular uptake of SYTOX Green (Figure 2I), release of lactate dehydrogenase (LDH) (Figure 2E), and decreased cell viability (Figure 2G), again in a GSDMD-dependent manner (Figure 2, F and H), proving pyroptotic cell death.

**Figure 3:**
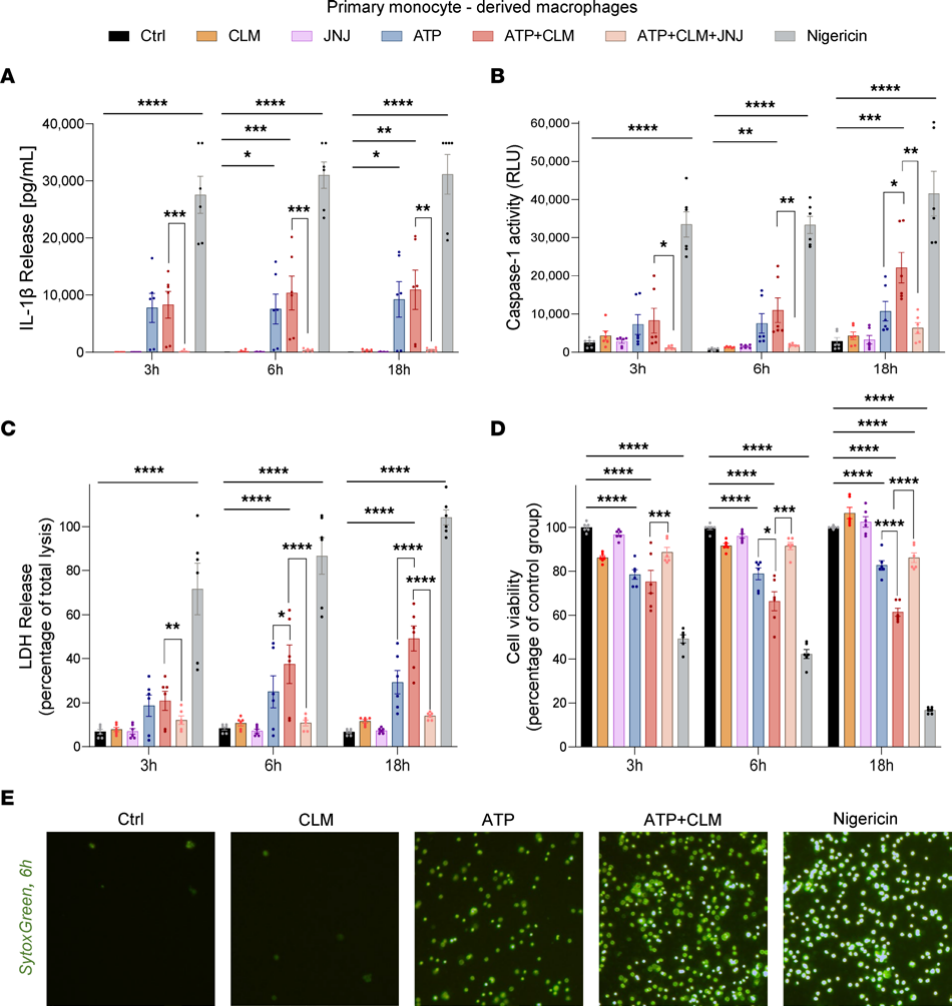
The potentiation effect of CLM on ATP-induced lytic cell death in primary monocyte-derived macrophages. The primary monocyte-derived macrophages (**A**–**D**) were primed with 200 ng/mL LPS overnight, then pretreated (JNJ and ATP+CLM+JNJ groups) with 30 nM JNJ-54175446 (a selective purine P2X7 receptor antagonist) for 1 hour, followed by treatment either with medium + DMSO (negative control, Ctrl), 10 μg/mL CLM, 2 mM ATP ± 10 μg/mL CLM, or 10 μM nigericin (positive control) for 3 hours, 6 hours, and 18 hours. Levels of the proinflammatory cytokine IL-1β (**A**) and caspase-1 (**B**) activity in the culture medium determined by ELISA and bioluminescence assay, respectively. Lytic cell death (**C**) determined by LDH activity released into the medium and cell viability (**D**) evaluated by the MTS assay. (**A**–**D**) Data are represented as mean ± SEM of 3 independent experiments, each performed in duplicate (*n* = 6). One-way ANOVA followed by Dunnett’s multiple comparisons test was used to compare the testing groups with control group (Ctrl). One-way ANOVA test followed by Holm-Šidák’s multiple-comparison was used to compare ATP and ATP+CLM group (to assess the potentiation effect of CLM) and ATP+CLM versus ATP+CLM+JNJ group. Panel **A**, nigericin at 3 hours (*n* = 2), 6 hours (*n* = 1), and 18 hours (*n* = 4) — above detection limit of the assay. *P ≤ 0.05; **P ≤ 0.01; ***P ≤ 0.001; ****P ≤ 0.0001. (**E**) Representative fluorescence microscopy images depicting SYTOX Green-stained MDMs treated with drugs for 6 hours. Original magnification, ×10.

Next, we confirmed these observations in primary human macrophages. Under identical conditions, in contrast to THP-1 cells, ATP alone caused lytic cell death in monocyte-derived macrophages, but the effect was further potentiated by CLM in a time-dependent manner (Figure 3, A–E). Compared with ATP alone, CLM significantly enhanced pyroptosis of macrophages assessed by an increased release of both active caspase-1 (Figure 3B and Supplemental Figure 1B) and LDH into culture supernatants (Figure 3C and Supplemental Figure 3, B, D, and F), increased cell membrane permeability (Figure 3E), and decreased cell viability (Figure 3D).

In contrast to THP-1 cells, primary human macrophages released higher quantities of IL-1β in response to ATP alone and CLM potentiated IL-1β release only marginally (Figure 3A and Supplemental Figure 3, A, C, and E).

To confirm that the potentiating effects of CLM on pyroptosis of primary macrophages were P2RX7 dependent, we pretreated macrophages with a selective brain-penetrant P2RX7 antagonist JNJ-54175446 (Figure 3, A–D) and observed significant attenuation of lytic cell death.

Next, we performed the bulk RNA-Seq analysis of primary human macrophages. Ingenuity Pathway Analysis (IPA) predicted inflammasome activation, pyroptosis, and immunogenic cell death (Supplemental Figures 4–7) as pathways affected by ATP+CLM treatment. We conclude that in the proinflammatory environment with extracellular ATP, CLM markedly enhances inflammasome activation and pyroptotic cell death of myeloid cells.

### CLM potentiates immunogenic cell death of human oligodendrocytes.

Intriguingly, oligodendrocytes (OLGs) express the highest *P2RX7* mRNA levels among human cell types, and the spinal cord has the highest *P2RX7* tissue expression (27, 28). Because the CLM-induced disability progression was clinically localized to the spinal cord, we hypothesized that OLGs are susceptible to pyroptosis in a proinflammatory environment with high extracellular ATP concentrations.

**Figure 4:**
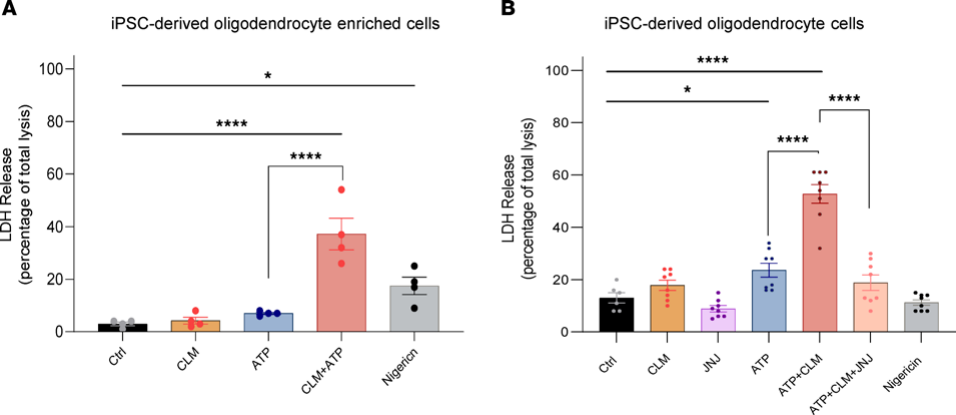
The potentiation effect of CLM on ATP-induced lytic cell death in iPSC-derived OLGs. iPSC-derived OLG-enriched cells (**A**) were primed with 200 ng/mL LPS overnight followed by treatment either with medium + DMSO (negative control, Ctrl), 10 μg/mL CLM, 2mM ATP ± 10 μg/mL CLM, or 10 μM nigericin (positive control). LDH release after the treatments for 18 hours. Data are represented as mean ± SEM; 1 donor with 4 technical replicates (*n* = 4). One-way ANOVA followed by Dunnett’s multiple-comparisons test was used to compare the testing groups with control group (Ctrl). One-way ANOVA test followed by Holm-Šidák’s multiple-comparison test was used to compare ATP and ATP+CLM group (to assess the potentiation effect of CLM). *P ≤ 0.05; ****P ≤ 0.0001. iPSC-derived OLG cells (**B**) were primed with TNF-α (50 ng/mL) overnight, pretreated with 60 nM JNJ-54175446 for 1 hour, followed by treatment either with medium + DMSO (negative control, Ctrl), 10 μg/mL CLM, 2 mM ATP ± 10 μg/mL CLM, or 2.5 μM nigericin. LDH release after the treatments for 18 hours. Data are represented as mean ± SEM of 2 independent experiments, each performed in triplicate (*n* = 6) for control or in 4 replicates for the other groups. One-way ANOVA followed by Dunnett’s multiple-comparisons test was used to compare the testing groups with control group (Ctrl). One-way ANOVA test followed by Holm-Šidák’s multiple-comparison test was used to compare ATP and ATP+CLM group (to assess the potentiation effect of CLM) and ATP+CLM versus ATP+CLM+JNJ group. *P ≤ 0.05. ****P ≤ 0.0001.

Thus, we differentiated human OLGs from induced pluripotent stem cells (iPSCs) using a published protocol (32) and tested to determine whether OLGs are susceptible to lytic cell death in the presence of extracellular ATP. We found that while insufficient alone, 2 mM ATP caused the lytic cell death of OLGs in the presence of CLM (Figure 4A).

As the iPSC differentiation protocol used in the above experiments leads to OLG-enriched cultures that also contain astrocytes (33), we sought to reproduce our results in pure OLGs. Using commercially available iPSC-derived OLGs (#1028, bit.bio), we confirmed that CLM added to sublytic doses of extracellular ATP causes immunogenic cell death, characterized by extracellular release of LDH. Similarly to primary monocytes, the CLM effect is dependent on P2RX7. Pretreatment of the cells with the P2RX7 antagonist JNJ-54175446 abrogated lytic cell death (Figure 4B). We conclude that in a proinflammatory environment with sublethal concentrations of extracellular ATP, CLM causes lytic cell death in OLGs. While pyroptotic cell death of OLGs is the most plausible mechanism of the observed CLM toxicity, it remains unclear whether pyroptosis contributes to the progression of MS disability in patients not treated with CLM.

### P2RX7 expression and the pyroptosis signaling pathway are increased in microglia and OLGs in MS brain tissue.

To investigate whether *P2RX7* expression associates with the transcriptional pyroptosis signature in MS CNS, we reanalyzed publicly available single-nucleus RNA-Seq (snRNA-Seq) datasets.

**Figure 5:**
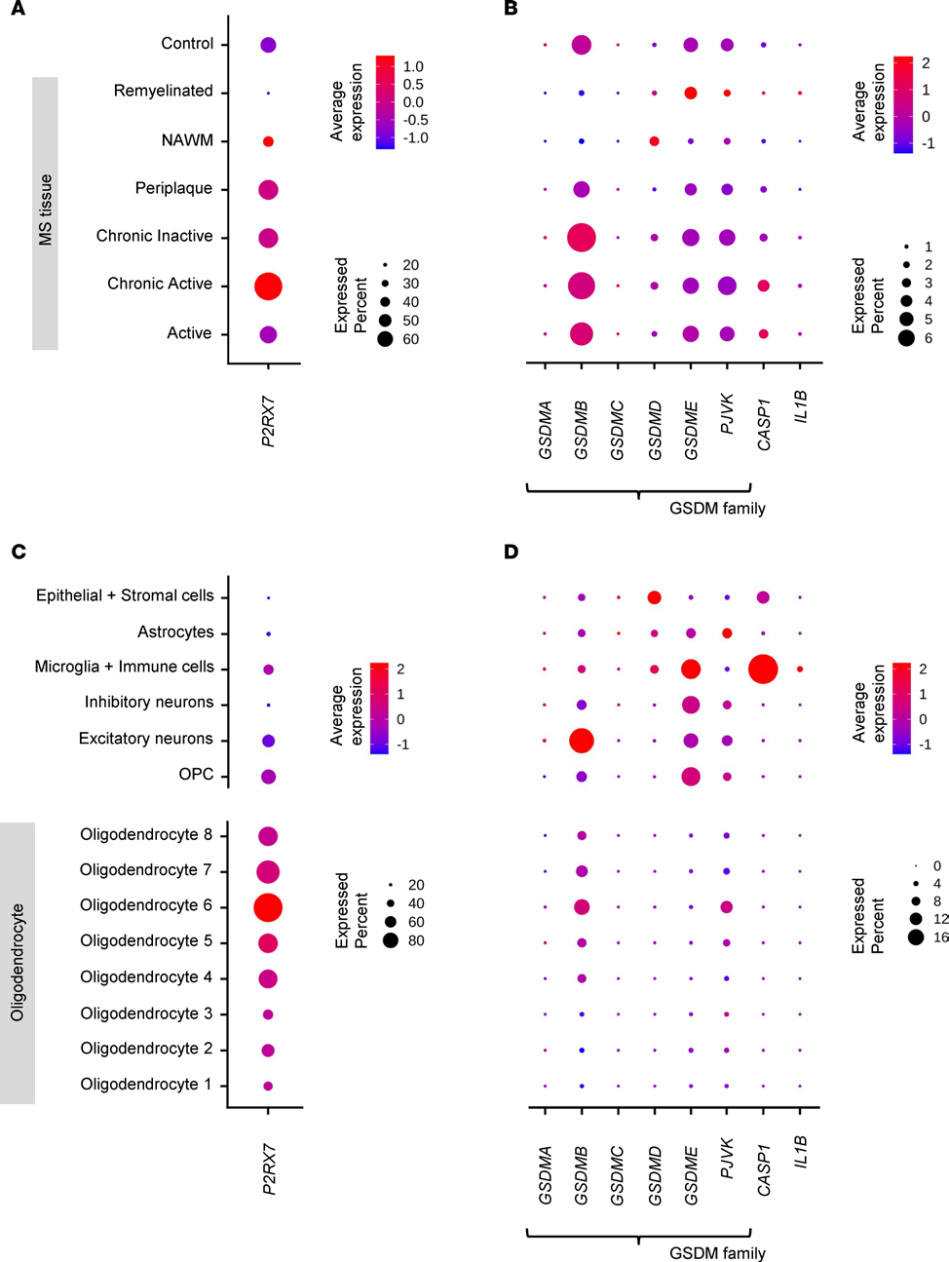
Gene expression associated with pyroptosis signaling pathway in CNS tissue. Published data of snRNA-seq from MS and control (GSE180759, GSE118257, and PRJNA544731) was reanalyzed, consisting of 21 MS subjects (2 PPMS and 19 SPMS) and 17 controls. Panels **A** and **C** illustrate the profiles of *P2RX7*, *GSDM* family, *CASP1*, and *IL-1β* across the lesion types. Meanwhile, panels **B** and **D** show gene expressions based on cell types.

We found enhanced *P2RX7* expression in MS compared with control brains (Figure 5A). Chronic active MS lesions, followed by periplaque white matter and chronic inactive lesions, had higher *P2RX7* expression than active lesions. We confirmed high expression of *P2RX7* in OLGs. Within the OLG subtypes, *P2RX7* was most highly expressed in the OLG6 subtype, which we found highly enriched in MS tissue (Figure 5B).

Assessing the spectrum of gasdermin channels, we found gasdermin B (*GSDMB*) most prevalent, especially in chronic active, chronic inactive, and active MS lesions (Figure 5C). Consistent with public databases, OLGs expressed *GSDMB* and pevjakin (*PJVK*) gasdermin channels. We found the highest expression of these in the aforementioned OLG6 subgroup, greatly expanded in MS compared with non-MS CNS (Figure 5D). On the other hand, *GSDMD* was expressed in microglia and epithelial/stromal cells and *GSDME* in microglia, neurons, and astrocytes, but at levels comparable between MS and control tissues (Supplemental Figure 8).

We integrated transcriptomic data for all known proteins involved in pyroptosis into a pyroptosis transcriptomic activation score (see Methods). The pyroptosis transcriptomics score was highest in microglia/macrophages (12.42 in MS versus 8.6 controls; 44% increase in MS), then epithelial and stromal cells (8.1 MS versus 7.88 controls), and finally in OLGs, where the pyroptosis score was 173% higher in MS compared with non-MS cells (4.69 MS versus 1.72 controls).

We conclude that MS microglia/macrophages and the particular subgroup of OLGs greatly expanded in MS have higher transcriptomic signatures of pyroptosis in comparison with control cells. Genes associated with pyroptosis show the highest transcription levels in chronic active MS lesions, followed by periplaque white matter and chronic inactive lesions.

### Pyroptosis signaling pathway measured by CSF proteins positively correlates with MS severity.

Compared with placebo, CLM therapy markedly activates intrathecal pyroptosis. To assess whether pyroptosis contributes to progression of neurological disability in non–CLM-treated MS patients, we calculated a proteomic pyroptosis score (see Methods for details) from CSF proteomic data measured by DNA aptamers (i.e., SOMAscan) in a cross-sectional cohort of 168 MS patients and in a cohort of 72 longitudinally followed MS patients (assigned to placebo arms of 2 double-blind clinical trials of progressive MS: ClinicalTrials.gov NCT00950248 (34) and NCT01212094) (18) and 49 HDs (for demographic data see Supplemental Table 1).

**Figure 6:**
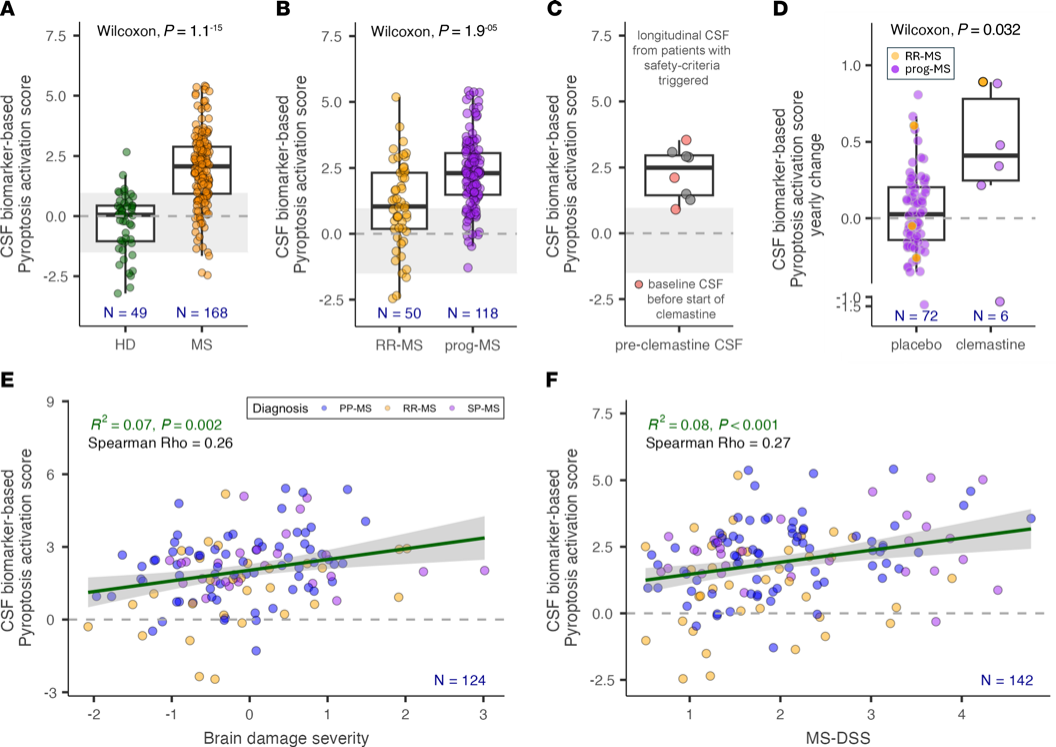
MS severity–associated pyroptosis signature in CSF increases with CLM treatment. CSF biomarker–derived pyroptosis activation score is significantly elevated in MS patients compared with HD (**A**) and in progressive MS (prog-MS) patients compared with RRMS patients (**B**). The gray area represents HD mean ± SD. (**C**) Longitudinal pre-CLM CSF samples of patients that triggered safety criteria show elevated pyroptosis activation score. CSF samples collected just prior to starting CLM are highlighted in red. (**D**) CLM-induced yearly change in pyroptosis activation score is significantly elevated compared with untreated MS patients. The lower and upper hinges of the boxplots correspond to the first and third quartiles (the 25th and 75th percentiles). The upper and lower whiskers extend from the hinge to the largest and smallest value, respectively. Pyroptosis activation scores are strongly associated with the rate of accumulation of cognitive and physical disability, represented by brain damage severity (**E**) and MS-DSS (**F**), respectively. The green line depicts linear regression line, and the gray shaded area shows 95% CI.

Consistent with our reanalysis of CNS tissue transcriptomics data, we observed a significant increase in the proteomic pyroptosis scores in MS versus HD CSF (*P* = 1.1 × 10^–15^, Figure 6A). Moreover, the CSF pyroptosis score was higher in progressive MS than in relapsing-remitting MS (RRMS) patients (Figure 6B, median 2.32 vs 1.23, *P* = 1.9 × 10^–5^) and was elevated in baseline (pretreatment) CSF samples in all 3 patients in the TRAP-MS CLM arm that triggered safety stopping criteria (Figure 6C). When comparing the yearly change in CSF pyroptosis score calculated from longitudinal CSF in MS patients assigned to the placebo arm of previous progressive MS trials with the CLM arm of TRAP-MS, we observed significant increases in CSF pyroptosis signatures in CLM-treated patients (Figure 6D, median 0.03 vs 0.41, *P* = 0.032).

Thus, we conclude that compared with healthy volunteers, MS patients have a CSF-proteomic signature indicative of intrathecal pyroptosis, that this signature is significantly (P = 1.9 ×10^–5^) higher in patients with progressive MS compared with RRMS, and that CLM further increases the intrathecal proteomic pyroptosis signature in vivo.

Furthermore, in the longitudinal MS cohort, the CSF pyroptosis activation score correlated significantly (Figure 6E; Spearman Rho = 0.26, R^2^ = 0.07, *P* = 0.002) with rates of brain tissue loss measured as brain damage severity score derived from volumetric MRIs (see Methods). Analogously, the CSF pyroptosis activation score also correlated with the MS disease severity score (MS-DSS; Figure 6F, Spearman Rho = 0.27, R^2^ = 0.08, *P* < 0.001), which integrates clinical and imaging data, reflects past, and predicts future rates of disability accumulation in the independent validation cohort (35).

Positive correlation of the CSF pyroptosis activation score with MS severity outcomes together with other presented results implicates pyroptosis as an important pathogenic mechanism of PIRA-associated CNS tissue destruction in MS.

## Discussion

In this study, we report that CLM increases the rate of disability accumulation in patients with advanced MS progressing by PIRA, by facilitating prolonged opening of the P2RX7 channel, potentiating intrathecal pyroptosis of myeloid cells and OLGs in MS CNS. In science, we quantify the probability of obtaining observed results by chance using *P* values. While in isolation every piece of data we present may be considered improbable but perhaps still possible by chance, collectively our data fit a unified story we find impossible to dismiss. Considering 2 obvious limitations of our study, that we: (a) do not have CNS tissue from CLM-treated subjects to demonstrate drug-induced oligodendroglial cell death and (b) did not run a clinical trial of the brain-penetrant P2RX7 inhibitor, which is the only way one could prove pathogenicity of P2RX7-mediated pyroptosis in MS, data we did collect provide robust practically obtainable evidence supporting our conclusions.

The strong counterargument is that CLM toxicity was not identified in the ReBUILD trial that included more MS patients. Indeed, we found this observation so reassuring that we attributed the unusual rates of disability progression in the first 2 safety criteria-triggering CLM arm patients to the weight gain from a sedentary lifestyle during the COVID-19 pandemic. But we knew we carefully selected safety criteria based on internal natural history data to uncover drug toxicity in MS progression with high sensitivity and accuracy. Furthermore, equally disabled patients treated in parallel with alternative drugs in the TRAP-MS platform trial did not experience analogous disability worsening. Therefore, after the third patient triggered the safety criterion, we stopped the CLM arm for toxicity in accordance with the approved protocol and focused on gaining a mechanistic understanding of this toxicity.

Designing the TRAP-MS trial with the belief that patients’ safety always takes precedence over scientific needs, we were doubtful that the resulting limited number of CLM-treatment CSF samples would elucidate toxicity mechanism(s). Instead, all of the data we obtained, both in vivo and in vitro, fitted like puzzle pieces into a unified story. After stopping the CLM arm, the first unexplained observations we presented to the TRAP-MS trial Data and Safety Monitoring Board were those of metabolic syndrome observed in CLM-treated subjects. Next, unbiased CSF proteomics showed enhanced purinergic signaling, observed only in CLM-treated patients. While our infectious disease colleagues were aware of the potentiating effect of CLM on P2RX7 ATP binding (25, 26) because they used CLM to enhance mycobacteria killing in human macrophages, none of the publications describing remyelinating effects of CLM identified or discussed this off-target effect, greatly relevant to MS. Likely nobody in the MS field, us included, knew about this potential CLM toxicity until we collected the data presented here.

By not activating P2RX7 directly, CLM may be toxic only in subjects with high CNS extracellular ATP levels. The rapid flow of vascular extravasate observed as dynamic contrast enhancement in acute MS lesions likely precludes focal accumulation of the extracellular ATP necessary to sustain P2RX7 signaling. This can also explain observations of Motawi et al. (36), where CLM attenuated pyroptosis and promoted remyelination in an experimental autoimmune encephalomyelitis (EAE) rat model. In contrast, in the smoldering inflammation associated with chronic active MS lesions or periplaque white matter, such focally high ATP levels may occur when OLGs are in the vicinity of activated myeloid cells and extracellular fluid flows slowly. This conclusion is in line with the first-in-human P2RX7 PET tracer study in 5 RRMS patients and 5 healthy controls that observed decreased binding of [^11^C]SMW139 in contrast-enhancing lesions, but increased binding in MS white matter (37).

This explains why we observed higher transcriptomics pyroptosis scores in chronic active/inactive MS lesions and periplaque white matter when compared with active MS lesions and why the CSF proteomic pyroptosis score was also significantly (*P* = 1.9 ×10^–5^) higher in progressive MS than RRMS. This also explains why much older (mean age of 62.5 versus 40.1 years) and more disabled (i.e., mean baseline EDSS 5.7 versus 2.2) TRAP-MS patients were more susceptible to the toxic effect of CLM compared with RRMS patients in the ReBUILD trial. CLM is highly lipophilic (26), which may increase its concentration in CNS white matter and in fat tissues, where it may aggravate metabolic syndrome. Our data suggest that preexisting obesity may be another susceptibility factor for CLM toxicity, perhaps by increasing inflammasome activation of adipose tissue macrophages and monocytes, which may travel to the CNS. The other differences between the ReBUILD and the TRAP-MS trials were treatment duration (2–3 months versus > 6 months) and more granular clinical scale to identify a safety signal in TRAP-MS (i.e., change in EDSS in ReBUILD versus change in CombiWISE slopes in TRAP-MS). Indeed, we show in Supplemental Figure 9, A–C, that using only changes in EDSS would not identify CLM toxicity.

In the recently published study, Cooper et al. (38) investigated the effect of CLM on demyelinated white matter lesions in a rabbit model that exhibits progenitor densities and limited remyelination, more closely resembling human MS tissues than traditional rodent models. The researchers found that chronic CLM treatment markedly reduced the pool of OLG progenitor cells (OPCs). Alongside the depletion, OPCs exhibited markers of senescence. While CLM can promote differentiation, its long-term use appeared to drive these progenitors to differentiate too rapidly, thereby depleting the reserve needed for sustained remyelination. Alongside the depletion, OPCs exhibited markers of senescence.

Considering that CLM fumarate is an over-the-counter antihistamine sometimes used off label based on the promising ReBUILD trial, our unfortunate experience highlights the potential for off-target effects of (most) small molecules. One cannot assume that a drug is safe for new indication because it is available over the counter. Likewise, safety cannot be extrapolated from studies that enrolled different patient populations, including different disease stages.

The silver lining in this story is that CLM toxicity helped to identify intrathecal P2RX7-linked pyroptosis as a likely PIRA mechanism even outside of CLM treatment. Although linking a CSF pyroptosis activation score to CNS pathology in living people is not possible, our data strongly suggest that MS patients with CSF proteomic pyroptosis signature are enriched for subjects progressing by PIRA and losing brain volume (i.e., brain damage severity score) without forming new MS lesions. In fact, most patients in the TRAP-MS trial did not form new MS lesions despite measurable disability progression on CombiWISE. The formation of new MS lesions triggers escalation of DMTs, because current DMTs suppress MS lesional activity at all MS stages (39). Thus, TRAP-MS patients are progressing by “nonlesional” MS activity (which encompasses PIRA) either because the MS lesion formation is fully suppressed by DMTs or because patients with advanced age stopped forming new lesions. Our retrospective application of the CSF proteomic pyroptosis score showed that 83% of these PIRA-experiencing patients who eventually received CLM (including 3 patients who triggered safety criteria) had an elevated CSF pyroptosis score before initiating CLM treatment.

Location of the brain transcriptional pyroptosis signature indicates that this lytic cell death occurs in MS tissue where OLGs are in the vicinity of smoldering inflammation characterized by activated microglia and macrophages. These myeloid cells undergo a vicious circle of inflammasome activation in response to inflammatory stimuli up to the point of succumbing to pyroptosis and releasing active caspase-1, IL-1β, IL-18, and DAMPs such as extracellular ATP and nuclear antigen HMGB1. Neighboring OLGs, having unusually high P2RX7 expression, open P2RX7 channels in response to extracellular ATP, releasing intracellular K^+^ and dying by immunogenic cell death/pyroptosis if the P2RX7 channel opening is prolonged, further fueling (sterile) inflammation. OLGs may be uniquely susceptible to P2RX7-mediated immunogenic cell death not only because of their high P2RX7 expression, but also because in contrast to most human cells, OLGs lack ATP-degrading ecto-nucleotidases NT5E (CD73) (40) and ENTPD1 (CD39) (41). Although our in vivo transcriptomic and proteomic assessment of pyroptosis pathways in MS and controls together with our in vitro data strongly imply a pyroptotic type of immunogenic cell death, we observed that human OLGs, both in healthy and MS CNS do not express GSDMD and GSDME channels. And although OLGs, especially the OLG6 subpopulation expanded in MS, express GSDMB and PJVK channels, lack of reagents and low numbers of human OLGs obtainable in vitro currently prevent formal proof that cleavage of GSDMB or PJVK proteins leads to formation of channels through which LDH is released. Nevertheless, our conclusions are supported by earlier pathology studies that identified inflammasome activation and pyroptotic cell death in microglia (42) and OLGs (43) in MS CNS. The current study makes 4 critical contributions: (a) it proposes that inhibiting P2RX7-mediated pyroptosis of OLGs is the desired effect of P2RX7 blockage in MS (as opposed to inhibiting inflammasome activation of microglial/macrophages); (b) it links intrathecal pyroptosis in living subjects to clinical and imaging outcomes of PIRA and MS severity; (c) it proposes that P2RX7 inhibitors may have a stronger therapeutic effect in progressive, compared with RRMS patients; and (d) it develops a CSF pyroptosis score that can identify patients with a therapeutic target and that can be used as pharmacodynamic (PD) marker in phase II human trials.

Our conclusion that P2RX7-mediated pyroptosis in OLGs is one of the candidate mechanisms of PIRA is supported by the informative cuprizone demyelination study (44), where P2RX7-deficient mice were robustly protected against toxic demyelination. On the other hand, density of myeloid cells in cuprizone demyelinated tissue was only marginally lower in P2RX7-deficient animals. Gene-expression profiling of microglia purified from demyelinated tissue showed that the proinflammatory phenotype of myeloid cells, including their expression of inflammasome-related genes, was not affected by the lack of P2RX7 (44). In view of our results, we reinterpret the results of this cuprizone study as supportive of the primary pathogenic role of P2RX7 signaling on OLGs, not on microglia. Indeed, direct triggering of P2RX7 in isolated rat optic nerve caused MS-like focal demyelinated lesions with associated axonal injury (45). Nevertheless, only OLG-specific P2RX7 deficiency could prove a primary pathogenic role of excessive P2RX7 signaling in in vivo demyelination.

An important question that neither our nor these animal studies addressed is, what physiological role does P2RX7 play in OLGs? Because P2RX7 expression is part of the myelin sheath organization cluster (28), long-term P2RX7 blockage may negatively alter myelin biology. Indeed, in rats, P2RX7 protein is present both in OLGs and compact myelin (visualized by electron microscope immunogold staining) (45). P2RX7 is also expressed in myelinated Schwan cells (46), where it affects developmental myelination of peripheral nerves. Reassuringly, blocking P2RX7 during the remyelination phase of the aforementioned cuprizone study did not alter remyelination and people with hypomorphic P2RX7 alleles do not have clinically relevant myelin pathology (see below).

The pathogenic role of P2RX7 in MS progression (and neurodegeneration in general) is also supported by *P2RX7* genetic polymorphism (47–49), where gain-of-function variants are observed at higher frequencies and hypomorphic variants at lower frequencies in different neurodegenerative diseases but also in faster aging (50). Nevertheless, the largest MS genetic study did not link P2RX7 polymorphism to MS severity (51). The heterogeneity and multiplicity of candidate pathogenic mechanisms (4, 22, 52, 53) and their evolution during MS natural history, compounded by inadequate accuracy of traditional MS severity outcomes, make studies of MS severity extremely challenging. Thus, this issue needs more research.

Proving pathogenicity of intrathecal pyroptosis outside of CLM toxicity requires an interventional clinical trial that successfully blocks pyroptosis in MS CNS. The pharmaceutical industry developed several P2RX7 inhibitors, some brain penetrant (e.g., JNJ-54175446 used in current study, GSK314181A, AZ11645373, AZD9056 and CE-224535) (54). P2RX7 inhibitors were tested in systemic autoimmune/inflammatory diseases such as rheumatoid arthritis and Crohn’s disease. Based on ClinicalTrials.gov, it seems that most P2RX7 inhibitors were abandoned after disappointing phase II trials (55, 56) or PD modeling (57). To our knowledge, only brain-penetrant JNJ-54175446, used in our in vitro experiments, is currently in clinical development for depression (58). The negative P2RX7 inhibitor trials are concerning, but they targeted the NLRP3 inflammasome, which has multiple/redundant activation modes. In contrast, we show that MS patients have increased intrathecal pyroptosis, which (for CSF proteomic pyroptosis score) correlates with rates of MS progression and is transcriptionally linked to P2RX7 expression in the subpopulation of OLGs greatly expanded in MS CNS. Furthermore, the high P2RX7 expression together with the lack of ecto-nucleases CD39 and CD73 makes OLGs uniquely vulnerable to P2RX7-mediated death. We hope our results will lead to a proof-of-principle clinical trial of the brain-penetrant P2RX7 inhibitor in MS. Preselecting patients using a CSF proteomic pyroptosis score and measuring drug-induced change in this CSF signature as a PD marker for rapid yes-no decisions would derisk more expensive trials, necessary to measure the effect of P2RX7 inhibition on PIRA.

## Methods

*Sex as a biological variable*. Both sexes were included in the study (for details, see *Subjects* section).

*Study design*. This study aimed to identify the CSF remyelination signature and collect the safety data on the long-term use of CLM in MS patients progressing by PIRA. Details of the trial design are described in Supplemental Figure 10 and the *Subjects* and *Sample processing* paragraphs below. For in vitro experiments, 3 independent experiments were conducted with 2 technical replicates to demonstrate biological reproducibility and ensure adequate statistical power for comparisons. The exception was the experiment for iPSC-derived OLGs, performed once (for iPSC-derived OLG-enriched cells) or twice (for commercial iPSC-derived OLGs) with 4 technical replicates. The figure legends indicate the group, sample sizes, and statistical tests used. No outliers or other data points were excluded.

*Subjects*. MS patients and HDs were prospectively recruited between January 2008 and January 2023 as part of the natural history protocol “Comprehensive multimodal analysis of neuroimmunological diseases of the central nervous system” (Clinicaltrials.gov NCT00794352). For details, see demographic data in Supplemental Table 1. All subjects underwent a comprehensive clinical examination, MRI, laboratory evaluation, and research lumbar puncture at the protocol entry, with optional follow-up visits. This protocol also served as a screening platform for identification of MS patients who would fulfill inclusion criteria for enrollment into the protocol TRAP-MS (ClinicalTrials.gov NCT03109288; for protocol design, see Supplemental Figure 10). Sixteen MS patients, fulfilling the inclusion criteria, were enrolled into the CLM arm of the TRAP-MS protocol. Nine of those completed at least 1 follow-up visit scheduled every 6 months. Forty-two patients were enrolled into an additional 6 treatment arms (dantrolene, hydroxychloroquine, losartan, montelukast, pioglitazone, and pirfenidone) of TRAP-MS. Both sexes were included in the study. In the TRAP-MS protocol, men and women represented similar proportions (Supplemental Table 1). Both male and female patients experienced worsening of disability on CLM therapy. CSF was collected at the baseline (within 1 year before treatment initiation) and 6 months after treatment start.

*Clinical outcomes*. Neurological exams were performed by an MS-trained clinician and recorded into NeurEx App (59) that automatically generates MS disability scores, such as EDSS. CombiWISE (24), brain damage severity (52), and MS-DSS (35) were calculated as described. Briefly, CombiWISE is a machine-learning–derived progression outcome that combines disability levels measured by EDSS, the Scripps Neurological Rating Scale (60), a timed 25-foot walk and, nondominant hand 9-hole peg test. Brain damage severity is calculated as age residuals of brain damage disability outcome that combines progression measured by cognitive test Symbol Digit Modalities Test (SDMT) (61) and MRI volumetric outcome brain parenchymal fraction (proportion of brain tissue volume to the whole brain volume). MS-DSS is a machine-learning–derived severity outcome that combines disability levels measured by CombiWISE, age, therapy history, and levels of CNS tissue destruction measured by MRI.

*Sample processing*. CSF was collected on ice and processed immediately according to a written standard operating procedure (62) by investigators blinded to diagnoses, clinical, and imaging outcomes. Aliquots were assigned alphanumeric identifiers and centrifuged for 10 minutes at 300*g* at 4°C within 30 minutes of collection. The cell-free supernatants were aliquoted and stored in polypropylene tubes at –80°C.

*SOMAScan analysis*. CSF protein content of MS patients and HDs was analyzed by SOMAScan technology (SomaLogic) using the version 4.1 of the assay that measures relative fluorescent units (RFUs) of 7,550 epitopes. The RFUs have been mathematically processed to normalize and calibrate the signal within and between different plates using control samples embedded in each of the 96-well plates.

### In vitro mechanistic studies

#### Reagents.

The reagents/growth factors used in the study are as follows: adenosine 5′-triphosphate disodium salt hydrate, ATP (MilliporeSigma, A6419), biotin (Sigma-Aldrich, 4639), CLM fumarate salt, CLM (Sigma-Aldrich, SML0445), insulin solution, human (Sigma-Aldrich, 19278), JNJ-54175446 (MedChemExpress, HY-117508), LPSs from *Escherichia coli* O111:B4 (Sigma-Aldrich, L4391), neurotrophin 3 (NT3) (EMD Millipore, GF031), nigericin sodium salt (Sigma-Aldrich, N7143), recombinant human IGF-I (R&D Systems, 291-G1-200), recombinant human HGF (R&D Systems, 294-HG-025), recombinant human macrophage colony stimulating factor (M-CSF) (PeproTech, 300-25), recombinant human PDGF (PDGF-AA) (R&D Systems, 221-AA-050), and 3,3,5-triiodo-l-thyronine (T3) (Sigma-Aldrich, T2877).

#### Cell cultures.

THP-1 and THP-1–KO–GSDMD cells were purchased from InvivoGen (thp-null and thp-kogsdmdz, respectively). The cells were grown in 1640 RPMI medium supplemented with 2 mM l-glutamine, 25 mM HEPES, 10% FBS (v/v), penicillin (100 U/mL) and streptomycin (100 μg/mL), and 100 μg/mL normocin at 37°C with 5% CO_2_. The THP-1–KO–GSDMD cells were maintained in a growth medium supplemented with the selective antibiotic 100 μg/mL zeocin following every other passage. Experiments were performed on the cells with less than 10 passages.

Monocytes elutriated from human peripheral blood were obtained from the NIH Blood Bank. Monocytes were cultured in RPMI 1640 medium supplemented with 10% FBS (v/v), 1% GlutaMax, penicillin (100 U/mL), and streptomycin (100 μg/mL). For differentiation to macrophages (MDMs), the cells were cultured for 7 days in the presence of recombinant human M-CSF (50 ng/mL).

Human iPSC-derived OLG-enriched cells were generated using a previously published protocol (30) in collaboration with the New York Stem Foundation. Neurospheres enriched for OLIG2 glia progenitors were plated into 96-well plates coated with poly-l-ornithine and laminin (one sphere per well) and cells were allowed to migrate out of the sphere. The cells were differentiated in PDGF medium containing morphogens that specifically promote OLG differentiation and maturation (DMEM/F12 medium containing nonessential amino acids [1×], GlutaMAX [1×], 2-mercaptoethanol, 100 U/mL penicillin, and 100 μg/mL streptomycin, supplemented with N2 supplement [1×], B27 supplement [1×], 10 ng/mL PDGF-AA, 10 ng/mL IGF-1, 5 ng/mL HGF, 10 ng/mL NT3, 60 ng/mL T3, 100 ng/mL biotin, 1 μM cAMP, 25 μg/mL insulin) for the next 40 days. Two-thirds of the medium was changed every other day.

Commercially available human iPSC-derived OLGs (io1028) were purchased from bit.bio and differentiated according to the manufacturer’s protocol. The cells were stabilized for 24 hours in comp:OM1 medium (DMEM/F12 containing B27 supplement [1×], N2 supplement [1×], GlutaMax [1×], nonessential amino acids [1×], 2-mercaptoethanol, 25 μg/mL insulin, 60 ng/mL T3, 100 ng/mL biotin, 1 μM cAMP, 20 ng/mL PDGF-AA, 5 ng/mL FGF-basic, 100 nM retinoic acid, 1 μM purmorphamine, 1 μg/mL doxycycline, and 5 μM ROCK inhibitor). Next the cells were differentiated for 8 days in complete maturation medium (DMEM/F12 containing B27 supplement [1×], N2 supplement [1×], GlutaMax [1×], nonessential amino acids [1×], 2-mercaptoethanol, 25 μg/mL insulin, 60 ng/mL T3, 100 ng/mL biotin, 1 μM cAMP, 5 ng/mL NT-3, 10 ng/mL IGF-3 and 1 μg/mL doxycycline). Half of the medium was changed every other day.

#### Inflammasome activation.

THP-1 and THP-1–KO–GSDMD cells were seeded in 96-well plates at 3 × 10^5^/well and primary MDMs at 1 × 10^5^/well and left for 4 hours. Then, for inflammasome activation, the cells were primed with 200 ng/mL LPS overnight, followed by activation with 10 μg/mL CLM, 2 mM ATP ± 10 μg/mL CLM, or nigericin (as positive control) for 3 hours, 6 hours, or 18 hours. We used 2.5 μM nigericin for THP-1 and THP-1–KO–GSDMD cells and 10 μM nigericin for primary MDM cells and iPSC-derived OLG-enriched cells. The cells primed with LPS without any activation treatment served as negative control. JNJ-54175446, a selective purine P2X7 receptor (P2X7R) antagonist at 30 nM, was used in the experiments involving primary MDM cells.

Human iPSC-derived OLG-enriched cells were generated using a previously published protocol (32). On day 70 of differentiation, iPSC-derived OLG-enriched cells were treated as above for 18 hours.

Human iPSC-derived OLGs (bit.bio) were primed on day 8 with TNF-α (50 ng/mL) overnight, then (where indicated) pretreated with 60 nM JNJ-54175446, followed by activation with 10 μg/mL CLM, 2 mM ATP ± 10 μg/mL CLM, or 2.5 μM nigericin for 18 hours. The cells primed with TNF-α without any activation treatment served as negative control.

After drug incubation, the conditioned medium was collected, centrifuged at 250*g* to remove cell debris, and either used immediately or frozen at –80°C until analysis. Inflammasome activation was evaluated by levels of the proinflammatory cytokine IL-1β and the activity of caspase-1 released to the cell culture medium, while pyroptotic cell death was assessed by SYTOX Green (Invitrogen) uptake, LDH release, or cell viability.

#### IL-1β release.

Quantification of secreted IL-1β was performed using the Human IL-1 Beta/IL-1F2 Quantikine ELISA Kit (R&D Systems, DLB50) according to the manufacturer’s instructions. THP-1 and THP-1–KO–GSDMD cell culture supernatants were diluted 2-fold, while MDM cell culture supernatants were 100-fold diluted. The samples were analyzed at 450 nm with a reference reading at 540 nm with the use of the Infinite 200 PRO microplate reader.

#### Caspase-1 activity.

After drug treatment, the culture medium was collected, centrifuged at 250*g* for 5 minutes to remove debris, and used immediately. Caspase-1 activity in cell culture supernatants was assayed using the Caspase-Glo 1 Inflammasome Bioluminescent Assay (Promega, G9952) per the manufacturer’s recommendations. Assay specificity was conferred by inclusion of a proteasome inhibitor, MG-132, in the lytic reagent and using a caspase-1 inhibitor, Ac-YVAD-CHO, at concentrations of 5 μM and 25 μM. Luminescence was recorded using the Promega Microplate Reader GloMax Explorer GM3500.

#### Activation of procaspase-1 and cleavage of GSDMD: Western blotting.

The PMA-differentiated THP-1 and *GSDMD*^–/–^ THP-1 cells were primed with 1 μg/mL LPS (4 hours) and then treated either with medium + DMSO (negative control, Ctrl), 10 μg/mL CLM, 5 mM ATP, 5 mM ATP+10 μg/mL CLM, or 2.5 μM nigericin (positive control) in FBS-free medium for 3 hours. After stimulation, cell culture supernatants and cell lysates were collected. Culture media were centrifuged at 250*g* for 5 minutes, to precipitate both floating cells and cell debris, and then concentrated 12.5× with Amicon Ultra 3K filters. Floating and adherent cells from the same well were pooled and lysed with RIPA buffer. The samples were subjected to SDS–PAGE and transferred to PVDF membranes (Bio-Rad). The membranes were blocked with 2.5%–5% BSA and then incubated overnight at 4°C with the following primary antibodies: GSDMD rabbit PolyAb (1:1000; Proteintech, 20770-1-AP, lot:00156359), caspase-1/p20/p10 rabbit PolyAb (1:1000; Proteintech, 22915-1-AP, Lot: 00154445) or β-actin antibody (C4), mouse monoclonal IgG1 (1:5000; Santa Cruz Biotechnology Inc., sc-47778/C4). Next, the membranes were incubated with goat anti-rabbit IgG (heavy chain) superclonal recombinant secondary antibody, HRP (1:10000; Invitrogen, A27036, Lot: 2961376). For β-actin, the membranes were incubated with IRDye 680RD goat anti-mouse IgG secondary antibody (1:5000; LICORbio, 926-68070, Lot: D40508-15).

#### Cell membrane permeability.

THP-1 cells were plated in ultra-low attachment, U-bottom plates, primed with 200 ng/mL of LPS overnight, and then stimulated either with medium + DMSO (Ctrl), 10 μg/mL CLM, or 2 mM ATP ± 10 μg/mL CLM in the presence of 12.5 nM SYTOX Green (Invitrogen, S7020) for 90 minutes. The SYTOX Green–stained cells were subjected to fluorescence analysis using 488 nm excitation on a flow cytometry Aurora 3 Laser V/B/R – 38 Channel System. MDM cells were plated in 35 mm glass bottom dishes, primed with 200 ng/mL of LPS overnight, and then stimulated either with medium + DMSO (Ctrl), 10 μg/mL CLM, 2 mM ATP ± 10 μg/mL CLM, or 10 μM nigericin for 6 hours. SYTOX Green (1μM) was added to the medium for monitoring cell membrane integrity. After 15 minutes, images of pyroptotic cells were captured using a Leica DMIL LED Fluorescence Microscope equipped with a green 488 nm laser at room temperature. The pictures were processed by Zeiss’ ZEN 2 (blue edition) software, version 3.6.095.01000.

#### Cell death assay.

Lytic cell death was assayed by using CytoTox 96 NonRadioactive Cytotoxicity Assay (Promega, G1780) according to the manufacturer’s protocol. The absorbance at 492 nm was measured as an indicator of LDH activity in cell culture supernatants with the use of an Infinite 200 PRO microplate reader (Tecan). The percentage of cytotoxicity was calculated with the following formula: percentage of cytotoxicity = 100 × (experimental sample − culture medium background)/(maximum LDH release − culture medium background).

#### Cell viability.

The cell viability was determined by CellTiter 96 AQueous One Solution Cell Proliferation Assay (Promega, G3580) as per the manufacturer’s instruction. Following treatment incubation, the medium was removed and replaced with 100 μL of fresh medium containing 20 μL of MTS reagent. After 2 hours incubation with MTS, the absorbance was measured at 490 nm using the Infinite 200 PRO microplate reader, and the percentage of viable cells was calculated using the following formula: percent of viability = 100 × (experimental sample)/(control sample).

### Bulk RNA-Seq

#### Sample preparation and RNA extraction.

For bulk RNA-Seq, the cells were plated in 6-well plates (1 × 10^6^ per well), primed with 200 ng/mL of LPS overnight, and then stimulated either with medium + DMSO (Ctrl) or 2 mM ATP ± 10 μg/mL CLM for 3 hours or 6 hours. For each drug condition, we sequenced the following samples (*n* = 3): (a) MDMs incubated with drugs for 3 hours; (b) MDMs incubated with drugs for 6 hours, and (c) THP-1 cells incubated with drugs for 3 hours. Total RNA extraction was performed using TRIzol LS reagent (Invitrogen). Three volumes of TRIzol LS reagent were added to each cell pellet (1 × 10^6^), lysate was combined with 200 μL of 1-bromo-3-chloropropane (MilliporeSigma), and the RNA aqueous phase was obtained according to the manufacturer’s recommendations (Thermo Fisher Scientific). RNA-containing aqueous phase was combined with 600 μL of RLT lysis buffer (QIAGEN) with 1% β-mercaptoethanol (MilliporeSigma), and RNA was extracted using the QIAGEN AllPrep DNA/RNA 96-well system. An additional on-column DNAse 1 treatment was performed during RNA extraction. The RNA integrity was verified with the Agilent RNA 6000 Pico kit on the 2100 Bioanalyzer (Agilent Technologies) according to the manufacturer’s protocol. RNA was quantitated using a fluorescence assay (Quant-it RiboGreen RNA, Thermo Fisher Scientific) on a Tecan Spark multiplate reader.

#### Library preparation and RNA-Seq.

The Stranded mRNA Prep Kit (Illumina) was used to generate sequencing libraries according to the manufacture’s reference guide #1000000124518, version 02. Briefly, 10 ng (sample 9-MDMs ATP+CLM 6 hours) or 25 ng (all others) total RNA was used as a template for library preparation, using 16 PCR cycles to enrich for adapter-modified products. Final libraries were pooled in equimolar concentrations and sequenced as paired-end 2 × 74 bp reads on 2 NextSeq 550 instrument runs using the High Output 150 Cycle Sequencing Kit (Illumina). Sequencing was performed on 2 NextSeq 550 (Illumina) 74 cycle plus 74 cycle symmetrical runs.

### Statistics

#### Identification of CLM-induced changes in CSF proteome.

The HD-scaled levels of somamers were compared before and 6 months after CLM treatment, and somamer changes outside of 95% of the normal distribution (mean ± 2 SD deviation) were evaluated by g:Profiler version e111_eg58_p18_30541362 to identify gene ontology terms and pathways that were significantly changed by CLM treatment. CLM-induced changes in somamer levels were also tested by 1-sample Wilcoxon’s test, with a null hypothesis of 0 change in somamer values between baseline and therapy sample. Both raw *P* values and FDR-adjusted *P* values were recorded.

#### CSF biomarker-based pyroptosis activation score calculation.

To calculate the pyroptosis activation score in CSF, first, we intersected known genes participating in pyroptosis signaling based on the knowledge base of the IPA platform with CSF proteins measured by the SOMAScan assay. Out of those, 10 proteins demonstrated statistically significant correlation with global MS severity outcome (sum of scaled values of brain damage severity [ref. 52], CombiWISE severity [ref. 52], and MS-DSS [ref. 35]) and were selected for the pyroptosis activation score calculation (Supplemental Table 3). The CSF pyroptosis activation score was then calculated as the sum of HD-scaled somamer levels of the selected 10 proteins, multiplied by the correlation coefficient of each protein with the global MS severity outcome.

#### In vitro mechanistic experiments.

Statistical analysis of data from in vitro mechanistic studies was performed using GraphPad Prism 9 (GraphPad Software Inc.). Data are represented as means ± SD or means ± SEM. One-way ANOVA followed by either Dunnett’s test (to compare various treatment groups with the control group) or Holm-Šidák’s test (to compare 2 experimental groups) was used, as indicated in figure legends. *P* < 0.05 was considered statistically significant.

#### Bulk RNA-Seq analysis.

Paired end FASTQ files were generated and concatenated. Preprocessing for FASTQ files (quality control, adapter trimming, quality filtering, per-read quality pruning of data) was performed by fastp package (63). The expression of transcripts was quantified based on GRCh38.p14 by using Salmon package (64). Preprocessing for FASTQ files and quantifying of gene expression were performed by cloud based Galaxy (version 0.23.2) (65). Raw reads count data were normalized, and comparison was performed by DESeq2 in iDEP 0.96 (66). K-means clustering principal component analysis (PCA) was done in iDEP. Significant gene expression change was defined as log_2_ fold change > 0.6 and FDR < 0.05. Data were analyzed using the QIAGEN’s IPA suite (QIAGEN, www.qiagen.com/ingenuity), spring 2023 release.

### Reanalysis of single-nucleus RNA sequencing

#### Published data collection.

Published data of snRNA-Seq from MS and control (NCBI’s Gene Expression Omnibus [GEO] GSE180759, GSE118257, and European Nucleotide Archive [ENA] PRJNA544731) were reanalyzed, consisting of a total of 21 MS subjects and 17 controls (age: 46.7 ± 6.9 versus 55 ± 14.5 years old, female proportion 10/21 [48%] versus 6/17 [35%], respectively). Mean MS disease duration was 19 ± 8.8 years and included 2 primary progressive MS (PPMS) and 19 secondary progressive MS (SPMS) patients.

#### Clustering and annotation of nucleus.

All of the raw data were counted, and quality control was checked by Cell Ranger, version 7.0 (10x Genomics). Count data reads were merged, and PCA and t-distributed stochastic neighbor embedding (tSNE) were analyzed by Seurat (version 4.2.0) (67). The nuclei with high mitochondrial gene content (> 10%) or high unique molecular identifier (UMIs) (> 10,000) were excluded, leaving 361,253 nuclei for downstream analyses. Each dataset was merged using canonical correlation analysis (Seurat, RPCA method). The merged dataset was clustered by Seurat, K-nearest neighbor (KNN) graph, and tSNE. Nonbiased clustering was done by weighted nearest neighbor analysis, and each cluster was annotated based on variable gene expression. The nucleus was classified as OPC, OLG, excitatory neurons, inhibitory neurons, microglia/immune cells, astrocytes, and epithelial/stromal cells. OLG were clustered into 8 subtypes (OLG1–8). Each gene expression was assessed based on (a) subject type (MS versus control), (b) lesion type (active lesion, chronic active lesion, chronic inactive lesion, control lesion, normal appearing white matter lesion [NAWM], peri plaque lesion, remyelinated lesion), and (c) clustered cell type.

#### Transcriptional pyroptosis pathway activation score calculation in snRNA-Seq.

To assess the cellular contribution to the pyroptosis pathway, we calculated transcriptional pyroptosis pathway activation (tPPAS) for each gene expression type. First, we downloaded all genes included in the pyroptosis pathway from IPA software, together with their directionality (i.e., transcription level correlating positively or negatively with pathway activation). For genes that lacked this directionality information, we used directionality of correlation coefficient of analogous CSF protein with global MS severity outcome (i.e., positive correlation coefficient of the CSF protein was translated as transcription level correlating positively with pathway activation). Next, we calculated average gene expression and percentage of cells expressing the gene for all genes in the pyroptosis pathway.

The linear regression model (*log_2_(1* *+* *y) ~ x*, where *x* = cell frequency expressing gene, and *y* = average gene expression of gene), was constructed to identify outliers (> 2SD or < –2SD of residual) that were excluded from further analysis. Finally, we calculated tPPAS of each cell type in MS and control tissue using the following formula:
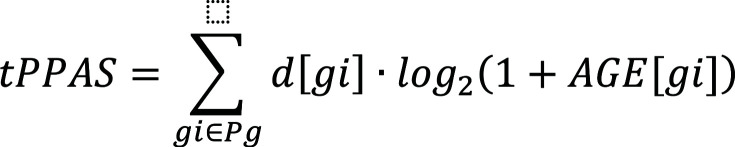


where *AGE* = average gene expression, *d* = directionality, *gi* = gene, and *Pg* = genes in pathway.

### Study approval

Written, informed consent was obtained from each patient. The clinical protocols were approved by the NIH Institutional Review Board.

### Data availability

Data supporting the findings of this study are included in the main article and Supporting Data Values file. All bulk RNA-Seq data that support the findings of this study are publicly available through NCBI’s Gene Expression Omnibus (GEO GSE268150). Reanalyzed published data of snRNA-Seq from MS and control can be found at the following: GEO GSE180759, GSE118257, and ENA PRJNA544731. This paper does not report custom code. Any additional information required to reanalyze the data reported in this paper is available from the corresponding author upon request.

## Author contributions

BB conceived the project. JK, PK, SA, and BB designed and conducted experiments and analyzed the data. RGM, KP, and BB contributed to the development of the methodology. BB, SMH, RGM, and KP provided the critical input to data interpretation. JK, PK, SA, and BB were responsible for data visualization and figure preparation. BB, KP, RGM, and VF supervised the project. BB acquired funding. NAP and VF provided resources. JK, PK, SA, and BB wrote the original draft of the manuscript. All of the authors reviewed the manuscript and took part in scientific editing.

## Supplementary Material

Supplemental data

Unedited blot and gel images

Supplemental table 2

Supplemental table 3

Supporting data values

## Version 1 05/15/2025

Electronic publication
