# Supplementary material for: Clemastine fumarate accelerates accumulation of disability in progressive multiple sclerosis by enhancing pyroptosis
Source: J Clin Invest. 2025 May 15;135(10):e183941. doi: 10.1172/JCI183941 (PMC12077908; doi:10.1172/JCI183941)
Supplement: Supplemental data [file jci-135-183941-s130.pdf]

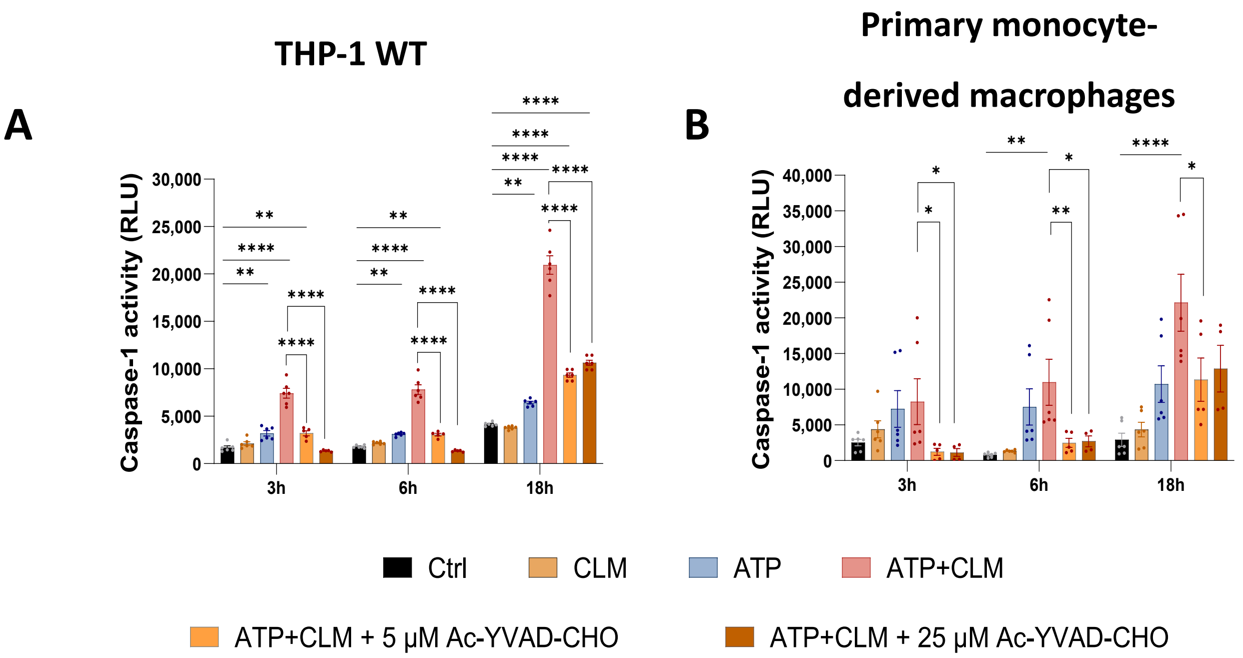

Fig. S1. Caspase-1 Assay Specificity. The cells were primed with 200 ng/mL LPS overnight followed by treatment either with medium + DMSO (negative control, Ctrl), 10 µg/mL CLM, 2 mM ATP, 2 mM ATP+10 µg/mL CLM with/without Ac-YVAD-CHO (5 µM and 25 µM) for 3h, 6h, and 18 h. Where indicated, the Ac-YVAD-CHO inhibitor was added to the lytic Caspase-Glo® 1 reagent to confirm the specificity of the luminescent caspase-1 signal. Activity of Caspase-1 was evaluated by bioluminescence assay in the cell culture supernatants of THP-1 cells (**A**) and primary human monocyte-derived macrophages (**B**). Data are presented as mean ± SD (THP-1 WT) and mean ± SEM (MDMs) (n=4-6). One way ANOVA followed by Dunnett's multiple comparisons test was used to compare the testing groups with control group (Ctrl). One-way ANOVA test followed by Holm-Sidak's multiple comparison was used to compare ATP+CLM and ATP+CLM with Ac-YVAD-CHO inhibitor group (to assess the Caspase-1 assay specificity). *, $P \leq 0.05$. **, $P \leq 0.01$. ***, $P \leq 0.001$. ****, $P \leq 0.0001$.

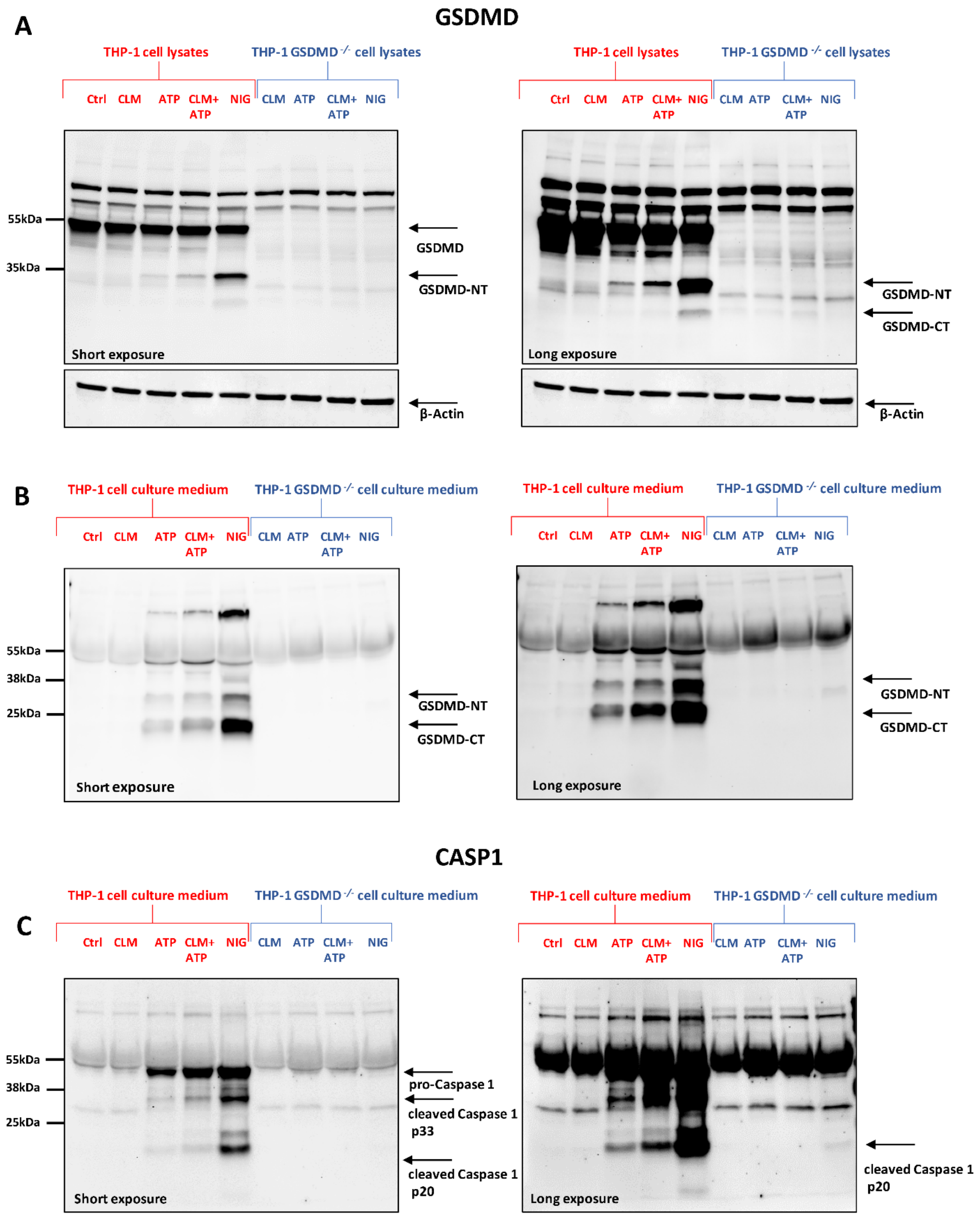
GSDMD
A
THP-1 cell lysates
THP-1 GSDMD -/- cell lysates
Ctrl CLM ATP CLM+ ATP NIG
CLM ATP CLM+ ATP NIG
55kDa
35kDa
GSDMD
GSDMD-NT
GSDMD-CT
Short exposure
Long exposure
β-Actin
B
THP-1 cell culture medium
THP-1 GSDMD -/- cell culture medium
55kDa
38kDa
25kDa
GSDMD-NT
GSDMD-CT
Short exposure
Long exposure
CASP1
C
THP-1 cell culture medium
THP-1 GSDMD -/- cell culture medium
55kDa
38kDa
25kDa
pro-Caspase 1
cleaved Caspase 1 p33
cleaved Caspase 1 p20
Short exposure
Long exposure

Fig. S2. The effect of Clemastine (CLM) on ATP-induced gasdermin D (GSDMD) cleavage and caspase 1 (CASP1) maturation. The PMA-differentiated THP-1 and $GSDMD^{-/-}$ THP-1 cells were primed with 1 µg/mL LPS (4h) and then treated either with medium + DMSO (negative control, Ctrl), 10 µg/mL CLM, 5 mM ATP, 5 mM ATP+10 µg/mL or Nigericin (2.5 µM, positive control) for 3h. After stimulation, cell lysates and/or cell culture supernatants (centrifuged at 250xg, 5min to remove the cell debris and then concentrated 12.5x with Amicon® Ultra 3K filters) were analyzed by Western blotting. The samples were subjected to SDS–PAGE and transferred to PVDF membranes, blocked with 2.5-5% BSA, and then incubated overnight at 4°C with the following primary antibodies: human GSDMD (20770-1-AP, 1:1,000; Proteintech) (**A&B**) or Caspase 1/p20/p10 Polyclonal antibody (22915-1-AP, 1:1,000; Proteintech) **(C)**. Next, the membranes were incubated with Goat anti-Rabbit IgG Secondary Antibody, HRP (1:10,000). For β-actin, the membranes were incubated with IRDye® 680RD Goat anti-Mouse IgG Secondary Antibody (926-68070, 1:10,000). GSDMD: full-length GSDMD; GSDMD-NT: N-terminal cleavage product of GSDMD (p30); GSDMD-CT: C-terminal cleavage product of GSDMD (p20), CASP1: pro-caspase 1, p20: 20 kDa cleaved caspase-1, p33: 33kDa cleaved caspase-1 (CARD-p20). Data are from 1 representative of 3 independent experiments with similar results.

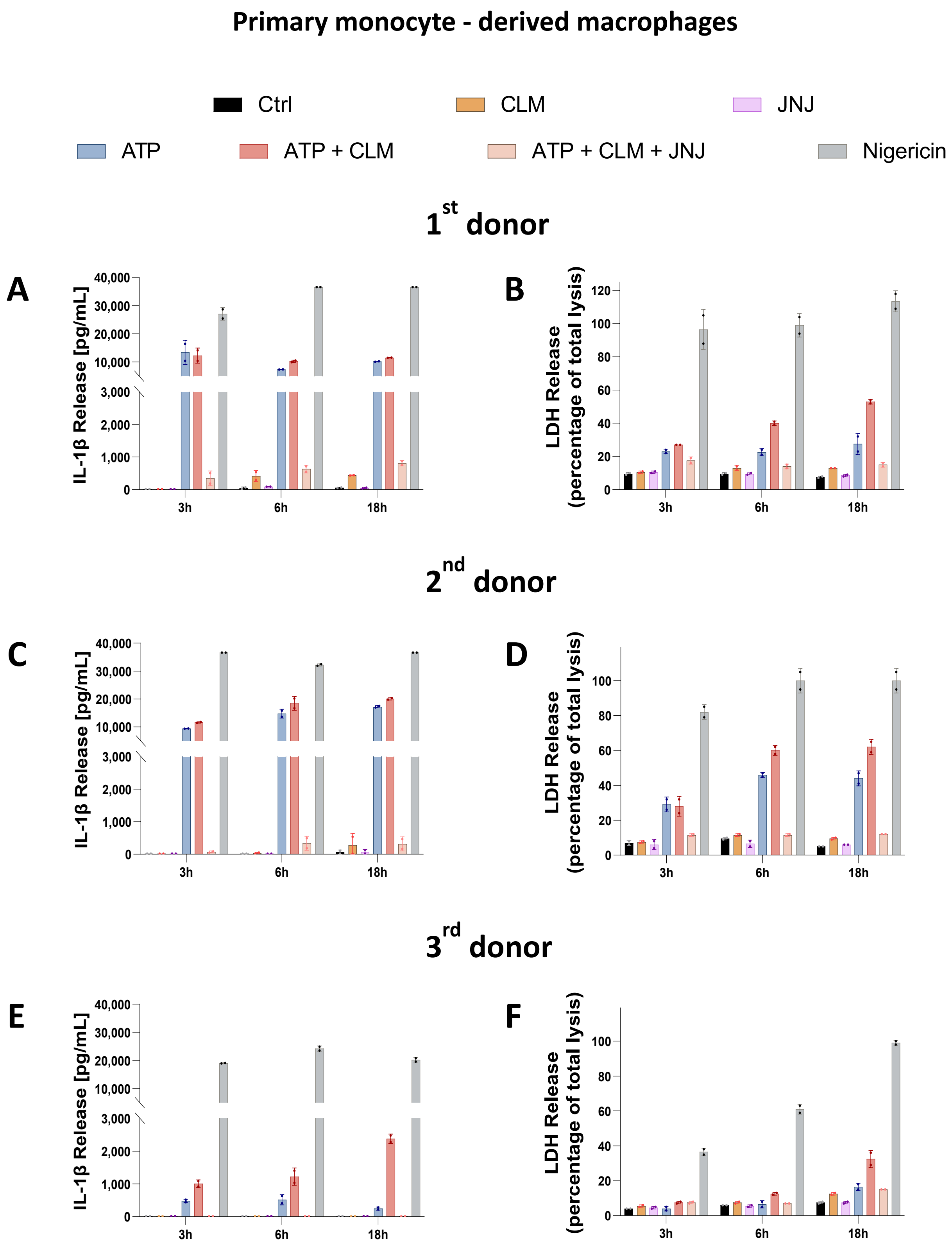
Primary monocyte - derived macrophages
Ctrl
CLM
JNJ
ATP
ATP + CLM
ATP + CLM + JNJ
Nigericin
1st donor
A
IL-1β Release [pg/mL]
40,000
30,000
20,000
10,000
3,000
2,000
1,000
0
3h
6h
18h
B
LDH Release (percentage of total lysis)
120
100
80
60
40
20
0
3h
6h
18h
2nd donor
C
IL-1β Release [pg/mL]
40,000
30,000
20,000
10,000
3,000
2,000
1,000
0
3h
6h
18h
D
LDH Release (percentage of total lysis)
100
80
60
40
20
0
3h
6h
18h
3rd donor
E
IL-1β Release [pg/mL]
40,000
30,000
20,000
10,000
3,000
2,000
1,000
0
3h
6h
18h
F
LDH Release (percentage of total lysis)
100
80
60
40
20
0
3h
6h
18h

Fig. S3. The effect of Clemastine (CLM) on ATP-induced pro-inflammatory cytokine IL-1β release and lytic cell death of primary monocyte-derived macrophages (MDMs) presented for each donor individually. The MDM cells were primed with 200 ng/mL LPS overnight, then pre-treated with 30 nM JNJ-54175446 (a selective purine P2X7 receptor antagonist) for 1 h, followed by treatment either with medium + DMSO (negative control, Ctrl), 10 μg/mL CLM, 2 mM ATP +/- 10 μg/mL CLM or 10 μM Nigericin (positive control) for 3h, 6h, and 18 h. Levels of pro-inflammatory cytokine IL-1β **(A,C,E)** and LDH activity **(B,D,F)** in the culture supernatants. Data are presented as mean ± SD of independent experiment performed in duplicate.

MYC Mediated Apoptosis Signaling

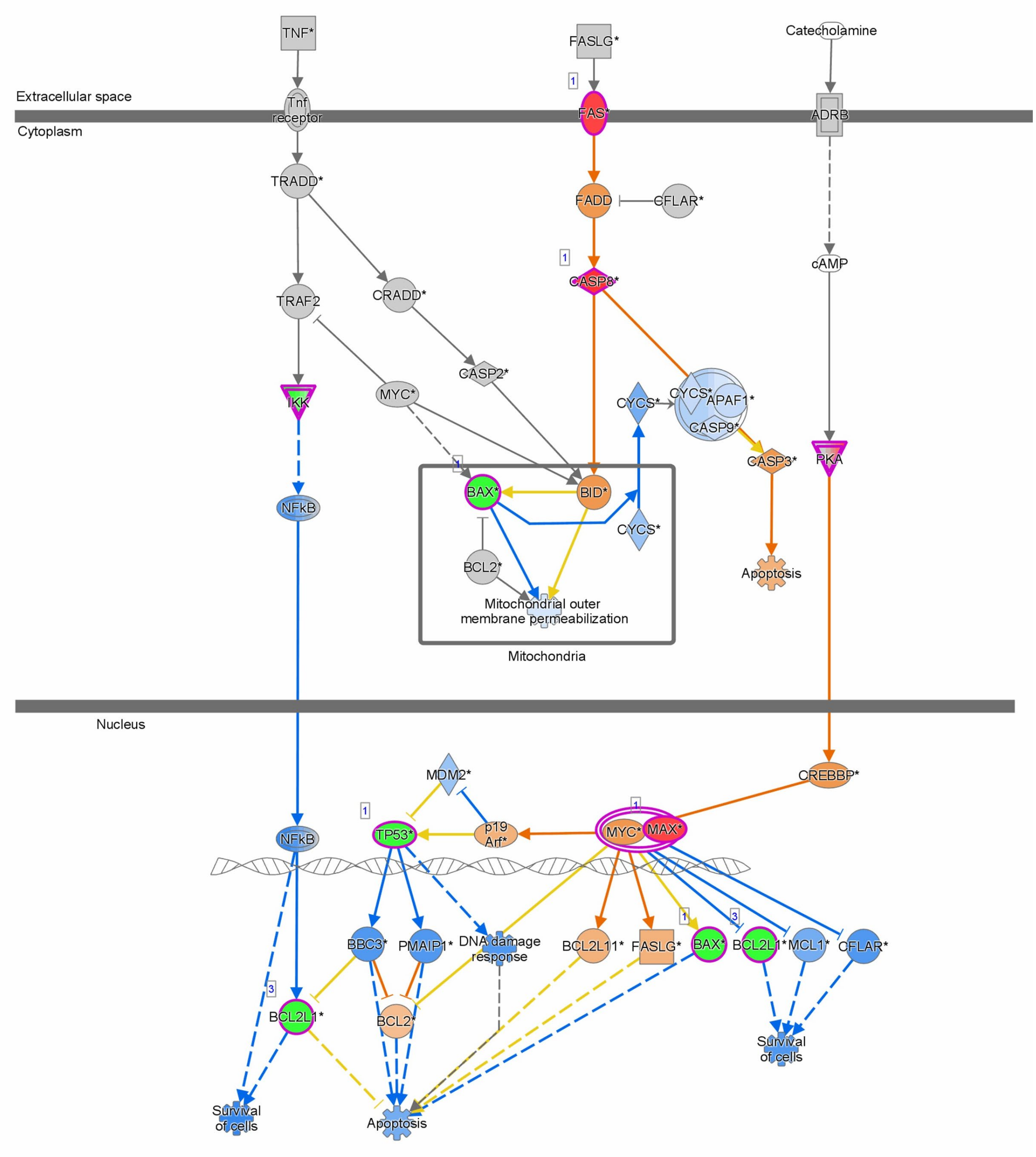

Fig. S4. Mitochondrial dysfunction and apoptosis pathways activated by ATP treatment predicted by ingenuity pathway analysis. mRNAs with FDR < 0.05 and log2 fold change > 0.6 was included in the activated pathways.

Inflammasome pathway

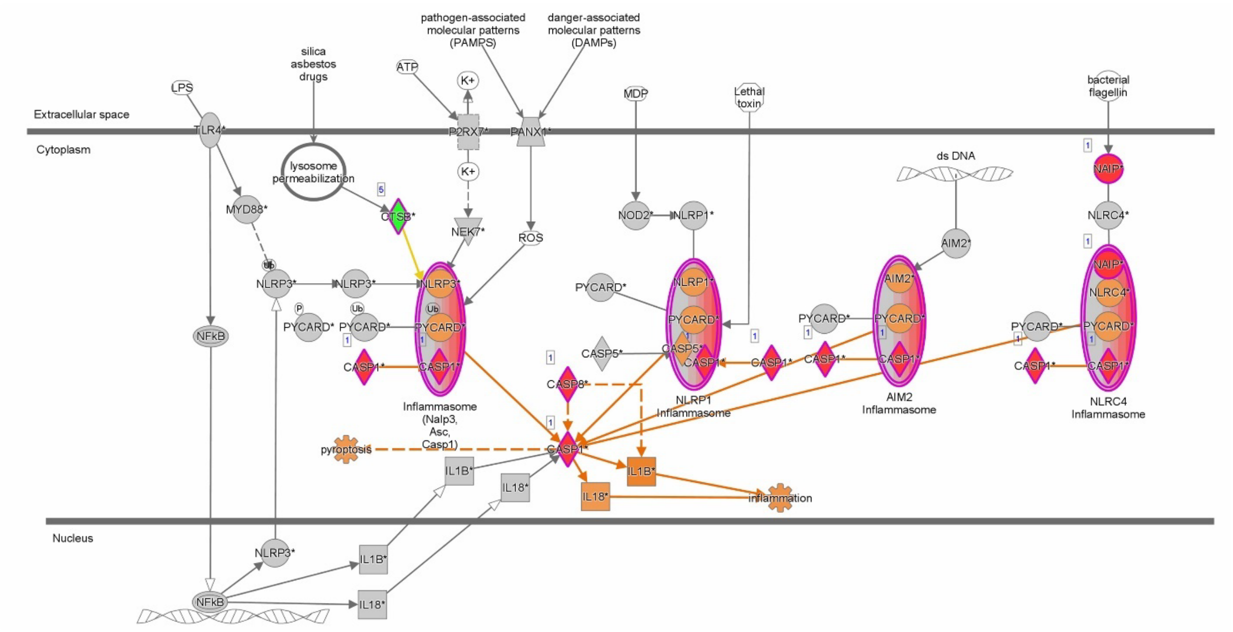

Fig. S5. The inflammasome signaling pathway activated by clemastine+ATP treatment predicted by ingenuity pathway analysis. mRNAs with FDR < 0.05 and log2 fold change > 0.6 was included in the activated pathways.

Pyroptosis Signaling Pathway

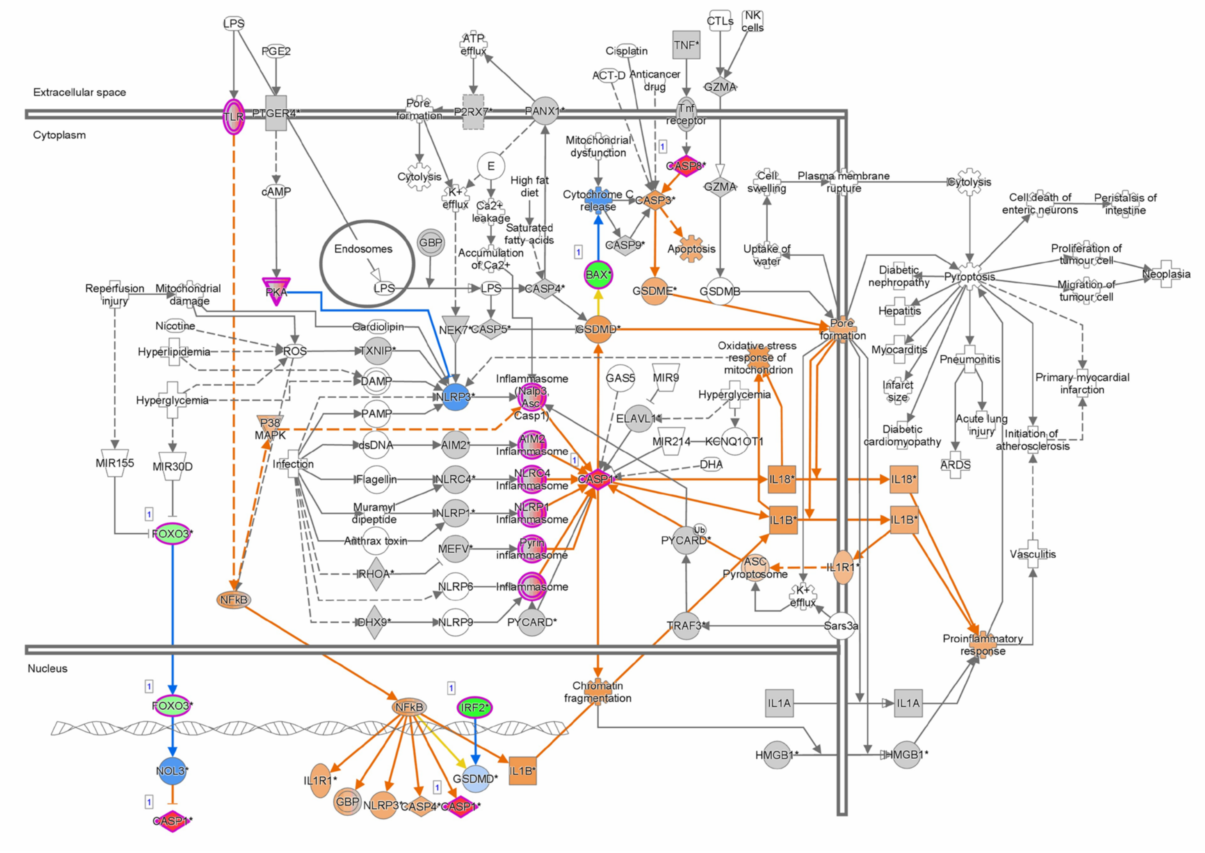

Fig. S6. The pyroptosis signaling pathway activated by clemastine+ATP treatment predicted by ingenuity pathway analysis. mRNAs with FDR < 0.05 and log2 fold change > 0.6 was included in the activated pathways.

Immunogenic Cell Death Signaling Pathway

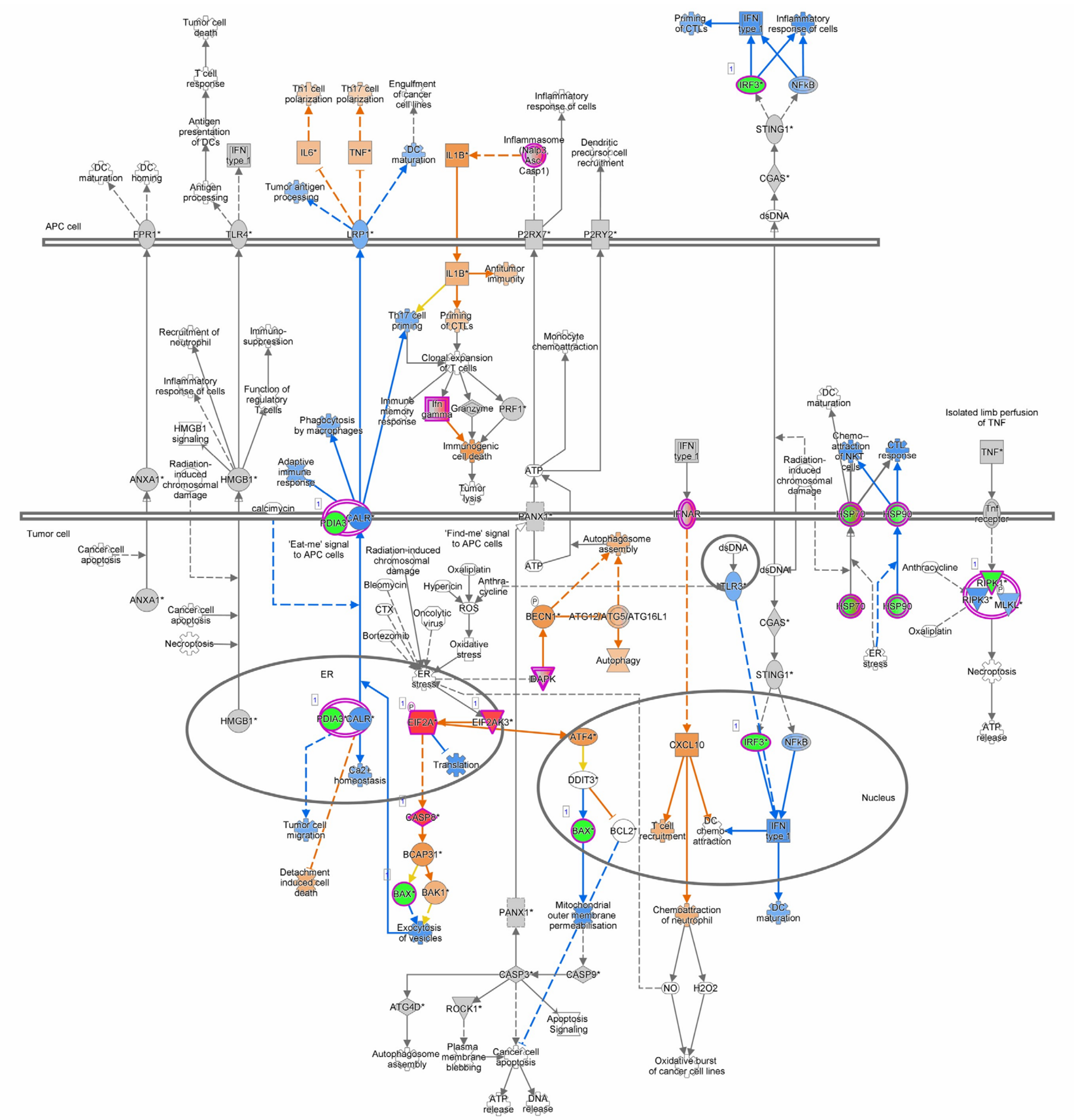

Fig. S7. The immunogenic cell death pathway activated by clemastine+ATP treatment predicted by ingenuity pathway analysis. mRNAs with FDR < 0.05 and log2 fold change > 0.6 was included in the activated pathways.

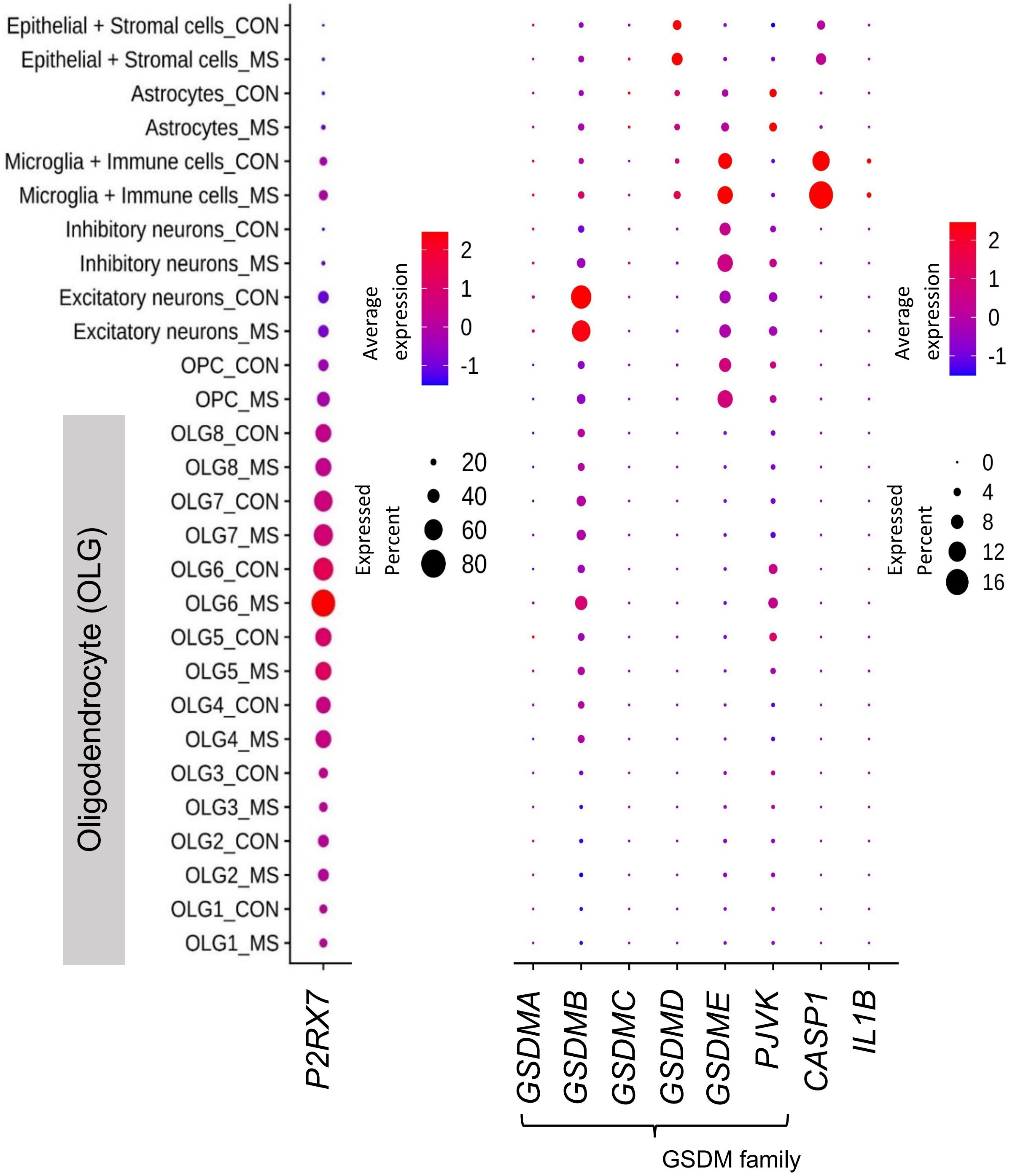

Fig. S8. **Gene expression associated with pyroptosis signaling pathway of CNS cell types: MS vs. control tissue**. Figure shows gene expressions based on cell types in MS (2 PPMS and 19 SPMS) compared to control tissue (CON, n=17). Expressed percent indicates the frequency of cells expressing the specific genes among all cells belonging to Y axis group. *P2RX7*: purinergic receptor P2X7; *GSDM:* gasdermin; *PJVK*: pejvakin; *CASP1*: caspase 1, *IL1β*: interleukin-1 beta; OPC: oligodendrocyte precursor cells, OLG: oligodendrocytes.

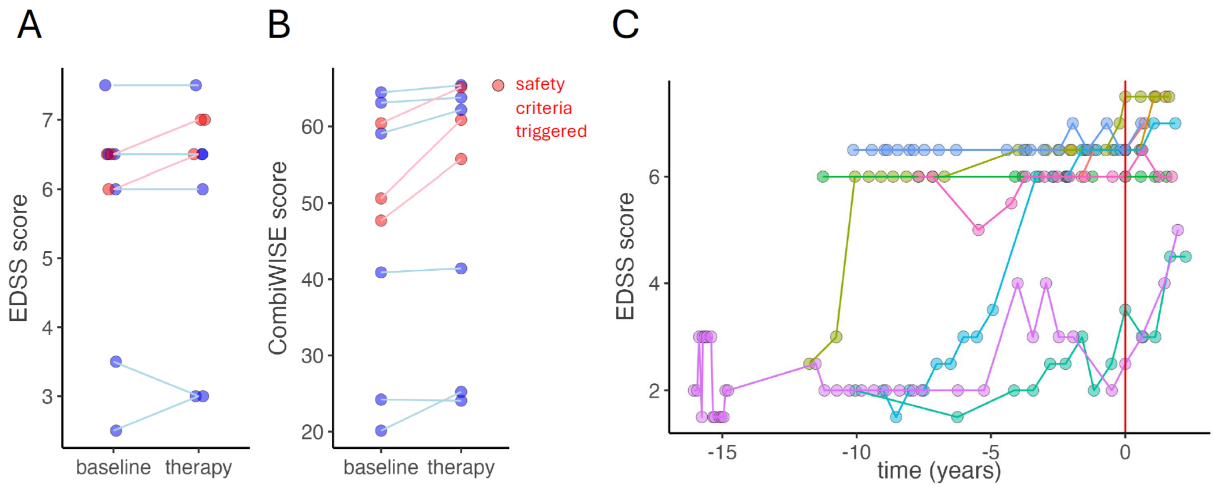

Fig. S9. Measurement of clinical disability in MS patients receiving clemastine treatment. Levels of clinical disability of nine patients treated with clemastine with at least one follow-up visit 6 months after therapy initiation were assessed by EDSS (**A**) and CombiWISE (**B**) at baseline (before clemastine initiation) and on therapy (6 month after clemastine start). Patients that triggered safety criteria are labeled in red. (**C**) Natural history EDSS data collected over >15 years; start of clemastine therapy is depicted with red vertical line.

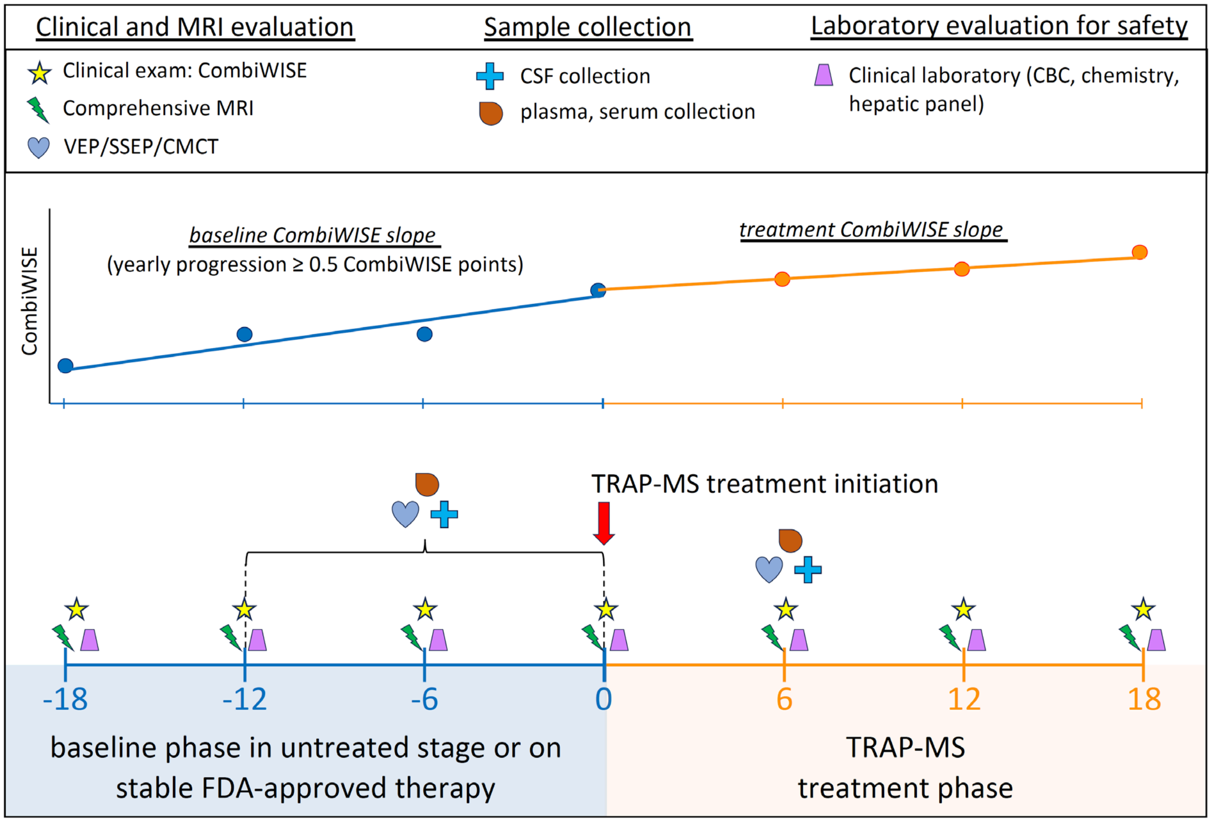

Fig. S10. Design of TRAP-MS trial. Patients were screened under the Natural History protocol for minimum of 18 months with minimum of 4 visits separated by at least 6 months on stable FD-approved therapy or untreated. At each visit, clinical exam, comprehensive MRI, and a panel of clinical laboratory tests were performed. Patients with calculated yearly CombiWISE change greater than qualified for TRAP-MS trial. Abnormal results from Visual Evoked Potential (VEP), Somatosensory Evoked Potential (SSEP) or Central Motor Conduction Time (CMCT) during baseline were required to start Clemastine as a TRAP-MS therapy. Baseline lumbar puncture (associated with plasma and serum collection) was performed within 12 months of TRAP-MS therapy initiation. After therapy initiation patients were followed every six months with clinical exam, MRI, and laboratory tests. Six months after therapy initiation an on-therapy lumbar puncture was performed (with associated plasma and serum collection) as well as electrophysiological test that was abnormal during the baseline. CombiWISE values on therapy were used to calculate therapy CombiWISE slope.

Table S1. Demographic data

Cohort		TRAP-MS clemastine	TRAP-MS other therapy	HD	Natural History cross-sectional	Placebo longitudinal
patients (N)		9	42	49	168	72
diagnosis (%)						
	RR-MS	22.2%	14.3%	NA	29.8%	4.2%
	PP-MS	33.3%	52.4%	NA	51.8%	15.3%
	SP-MS	44.4%	33.3%	NA	18.5%	80.6%
sex (%)						
	F	55.6%	50.0%	46.9%	58.3%	52.8%
	M	44.4%	50.0%	53.1%	41.7%	47.2%
Age (years)						
	mean	62.5	58.9	38.5	53.2	57.5
	SD	12.1	8.5	13.5	12.1	7.3
	range (min-max)	35.1-73.3	36.2-76.5	19.4-71.3	19.5-74.9	38.4-74.6
Disease duration (years)						
	mean	22.3	18.5	NA	13.9	15.3
	SD	11.8	12.3	NA	10.9	10.4
	range (min-max)	8.1-48.2	5.3-48.1	NA	0.1-54.1	0.1-42.2
EDSS						
	mean	5.7	5.4	0.9	5.0	5.3
	SD	1.6	1.4	0.9	1.7	1.6
	range (min-max)	2.5-7.5	2.0-7.0	0.0-3.0	1.5-8.0	1.5-8.0
