# Supplementary material for: Clemastine fumarate accelerates accumulation of disability in progressive multiple sclerosis by enhancing pyroptosis
Source: J Clin Invest. 2025 May 15;135(10):e183941. doi: 10.1172/JCI183941 (PMC12077908; doi:10.1172/JCI183941)
Supplement: Unedited blot and gel images [file jci-135-183941-s131.pdf]

Figure S2 A. Gasdermin D in cell lysates of THP-1 and THP-1 GSDMD KO cells, short exposure

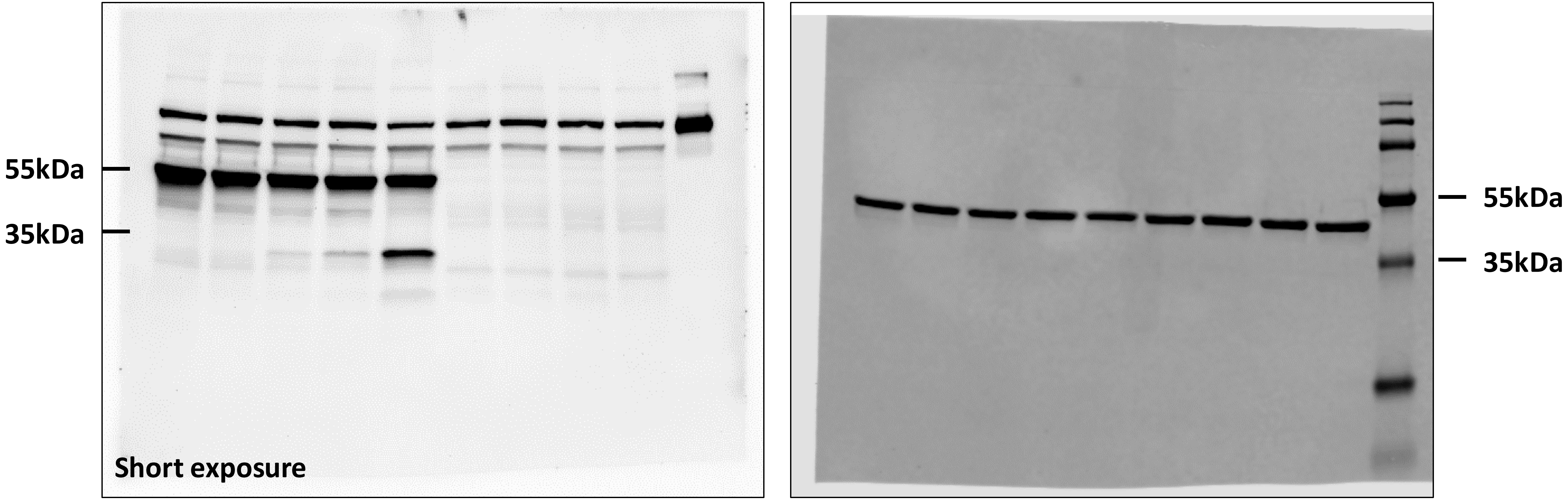

From left side: 1. Ctrl; 2. CLM; 3. ATP; 4. CLM+ATP; 5. Nigericin; 6. CLM; 7.ATP; 8. CLM+ATP; 9. Nigericin; 10. Ladder;

THP-1 cell lysates, THP-1 GSDMD KO cell lysates. Except for the ladder, the entire blot is presented in Fig. S2A.

Figure S2 A. Gasdermin D in cell lysates of THP-1 and THP-1 GSDMD KO cells, <u>long exposure</u>

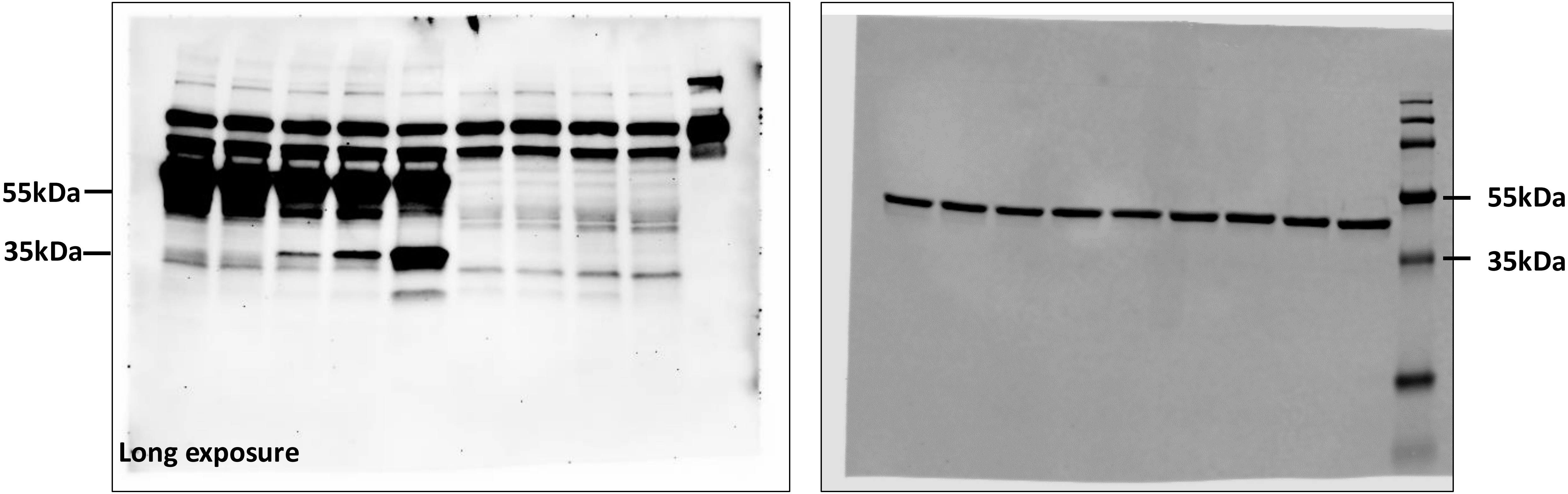

<u>From left side:</u> 1. Ctrl; 2. CLM; 3. ATP; 4. CLM+ATP; 5. Nigericin; 6. CLM; 7.ATP; 8. CLM+ATP; 9. Nigericin; 10. Ladder;

THP-1 cell lysates, THP-1 GSDMD KO cell lysates. Except for the ladder, the entire blot is presented in Fig. S2A.

Figure S2 B. Gasdermin D in culture medium of THP-1 and THP-1 GSDMD KO cells, short & long exposure

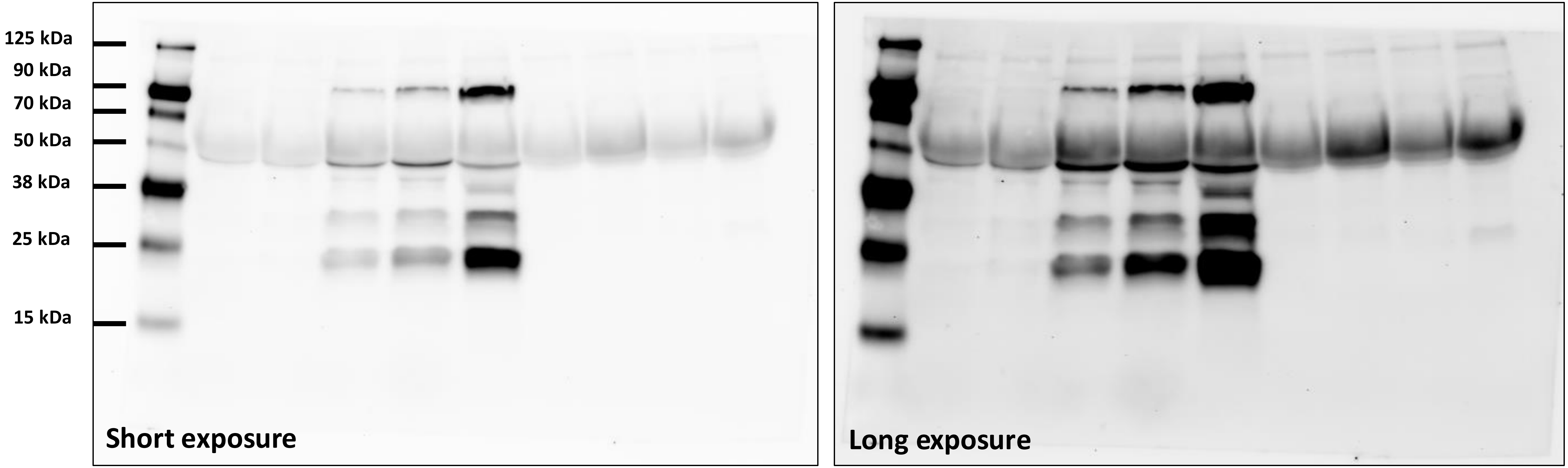

From left side: 1. Ladder; 2. Ctrl; 3. CLM; 4. ATP; 5. CLM+ATP; 6. Nigericin; 7. CLM; 8. ATP; 9. CLM+ATP; 10. Nigericin

THP-1 culture medium, THP-1 GSDMD KO culture medium. Except for the ladder, the entire blot is presented in Fig. S2B.

Figure S2 C. Caspase 1 in culture medium of THP-1 and THP-1 GSDMD KO cells, short & long exposure

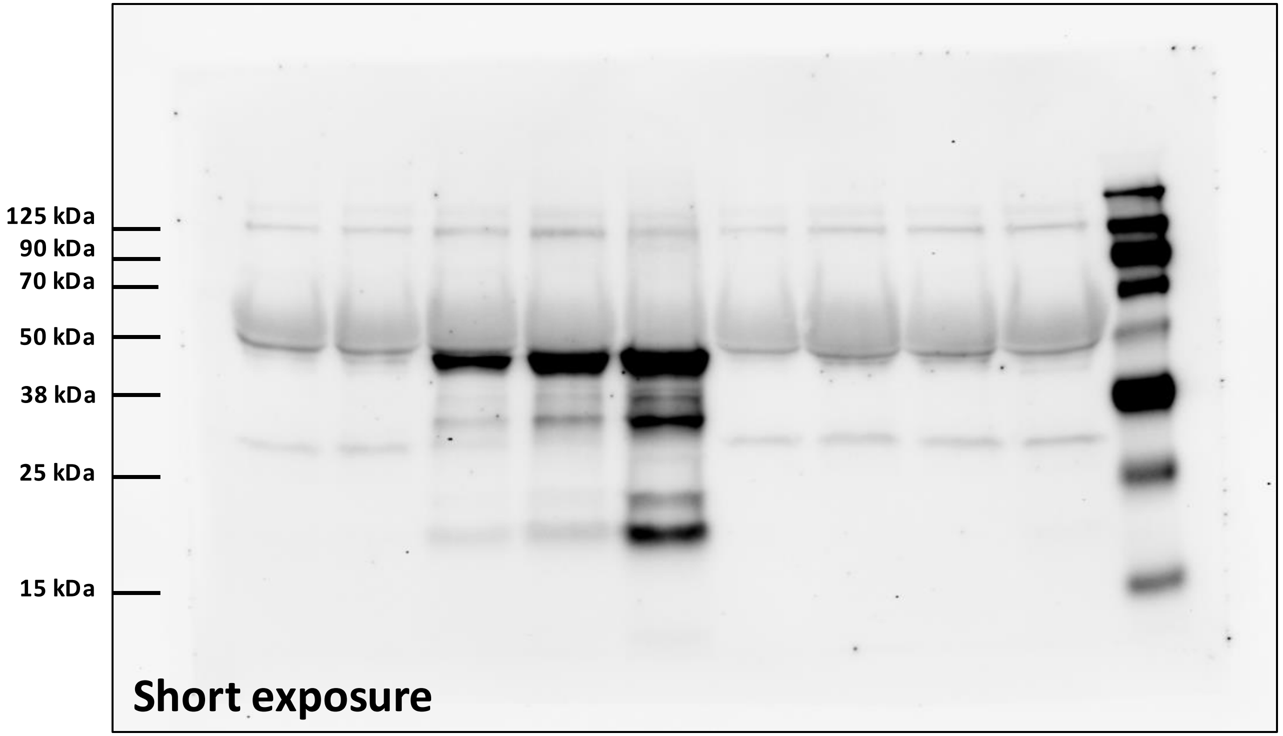

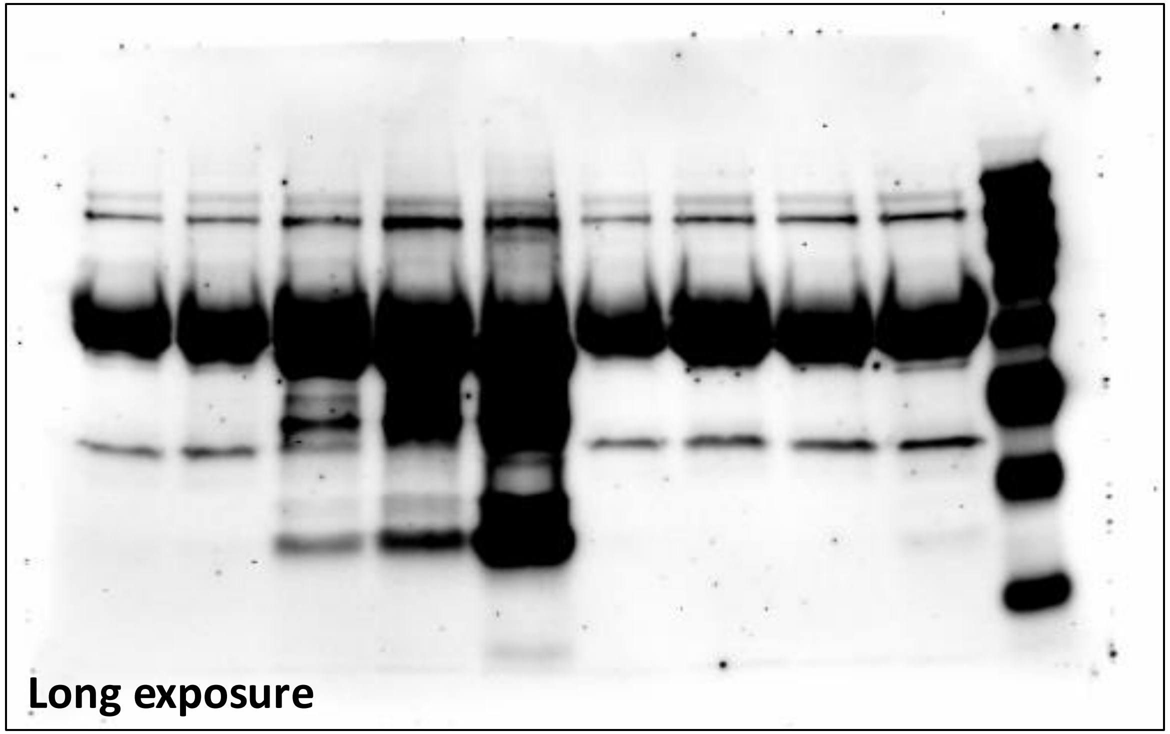

From left side: 1. Ctrl; 2. CLM; 3. ATP; 4. CLM+ATP; 5. Nigericin; 6. CLM; 7.ATP; 8. CLM+ATP; 9. Nigericin; 10. Ladder;

THP-1 culture medium, THP-1 GSDMD KO culture medium. Except for the ladder, the entire blot is presented in Fig. S2C.
